# LC3-positive structures are prominent in autophagy-deficient cells

**DOI:** 10.1038/s41598-019-46657-z

**Published:** 2019-07-12

**Authors:** Gautam Runwal, Eleanna Stamatakou, Farah H. Siddiqi, Claudia Puri, Ye Zhu, David C. Rubinsztein

**Affiliations:** 1 Department of Medical Genetics, Cambridge Institute for Medical Research, The Keith Peters Building, Cambridge, CB2 0XY UK; 2 UK Dementia Research Institute, The Keith Peters Building, Cambridge Biomedical Campus, Hills Road, Cambridge, CB2 0XY UK

**Keywords:** Cellular imaging, Macroautophagy

## Abstract

Autophagy is an evolutionarily conserved process across eukaryotes that degrades cargoes like aggregate-prone proteins, pathogens, damaged organelles and macromolecules via delivery to lysosomes. The process involves the formation of double-membraned autophagosomes that engulf the cargoes destined for degradation, sometimes with the help of autophagy receptors like p62, which are themselves autophagy substrates. LC3-II, a standard marker for autophagosomes, is generated by the conjugation of cytosolic LC3-I to phosphatidylethanolamine (PE) on the surface of nascent autophagosomes. As LC3-II is relatively specifically associated with autophagosomes and autolysosomes (in the absence of conditions stimulating LC3-associated phagocytosis), quantification of LC3-positive puncta is considered as a gold-standard assay for assessing the numbers of autophagosomes in cells. Here we find that the endogenous LC3-positive puncta become larger in cells where autophagosome formation is abrogated, and are prominent even when LC3-II is not formed. This occurs even with transient and incomplete inhibition of autophagosome biogenesis. This phenomenon is due to LC3-I sequestration to p62 aggregates, which accumulate when autophagy is impaired. This observation questions the reliability of LC3-immunofluorescence assays in cells with compromised autophagy.

## Introduction

Autophagy is a process that has been widely implicated in the pathogenesis of various conditions, such as neurodegenerative diseases, cancer, and inflammation. It targets substrates like long-lived proteins, aggregate-prone proteins, and damaged organelles for lysosomal degradation to maintain cellular homeostasis^1,2^. This process is conserved across eukaryotes and involves the formation of double-membraned structures known as autophagosomes. The double-membraned autophagosomes form from cup-shaped structures known as phagophores and are responsible for the engulfment of cargoes that are subsequently degraded after fusion with lysosomes^1^.

Autophagosome formation involves two successive ubiquitin-like reactions. The first reaction employs the E1-like ATG7 and the E2-like ATG10 enzymes, which conjugate the ubiquitin-like ATG12 to ATG5. This conjugate then forms a complex with ATG16L1^3^. The second set of reactions involves the ubiquitin-like LC3 protein family. LC3-I is generated by proteolytic cleavage of pro-LC3 by ATG4, which exposes a C-terminal glycine that is amenable to conjugation. ATG7, the E1-like enzyme, ATG3, an E2-like enzyme, and the ATG5-12-16L1 complex as the E3-like enzyme then conjugate LC3 family members to phosphatidylethanolamine (PE) on the surface of nascent autophagosomes^1^. The lipidated LC3, known as LC3-II, has a faster mobility than LC3-I on SDS PAGE and is relatively specifically associated with autophagosomes and autolysosomes (in the absence of conditions stimulating LC3-associated phagocytosis).

The autophagic system also harnesses proteins known as autophagy receptors, which increase the selectivity of the autophagic process by facilitating the engulfment of certain cargoes by the growing autophagosomes^4^. The autophagy receptor proteins share a common domain organisation containing both a ubiquitin-binding domain (UBD) and an LC3-interacting region (LIR)^1,4^, which allow them to act as bridging molecules recognising the degradation signal on the autophagic cargo on the one hand, and binding LC3 on the growing autophagosomal membrane on the other. The most widely studied autophagy receptor shown to play an important role in autophagy is p62/SQSTM-1 (sequestosome-1)^4,5^. As these molecules are themselves autophagy substrates, their levels often increase when autophagy is perturbed^5,6^.

Autophagosome numbers are widely assessed by quantifying LC3-II puncta numbers in cells using immunocytochemistry/immunohistochemistry for endogenous LC3, or immunofluorescence for fluorescent-tagged LC3^7,8^. These types of assays have been used for chemical and genetic screens and for assessing autophagy status both in cells and *in vivo* in different conditions. With such assays, the number or the overall area of LC3-positive structures can increase, if there is increased autophagosome formation or impaired degradation. However, it has been assumed that the numbers of LC3-positive structures and their area should decrease if autophagosome biogenesis is impaired and that there should be no LC3-positive structures in cells where LC3-II cannot form. Surprisingly, we find that these assumptions are incorrect, as we show that LC3-I can be associated with p62-positive aggregates, which are known to accumulate and form inclusion bodies when autophagy is compromised^6,9^. However, while the existing literature shows the formation of LC3-positive structures upon GFP-LC3 overexpression in autophagy-null cells from mice (MEFs)^9–12^, we show that endogenous LC3-I in ATG9- and ATG16L1-knockout cells of human origin (HeLa cells) forms distinct ubiquitin-positive aggregates in association with p62. More importantly, we show that this phenomenon occurs even under partial autophagy-deficient conditions after transient knockdown of essential autophagy genes, such as ATG7 and ATG10. The conditions of transient autophagy-deficiency in cells that occurs when using siRNA-mediated depletion also forms the underlying basis for many experimental strategies, such as drug screening assays in the field of autophagy. Therefore, these data have critical implications for interpreting one of the most widely used set of autophagy assays.

## Results

### Autophagy-deficient cells show aberrant LC3-positive puncta

**Figure 1:**
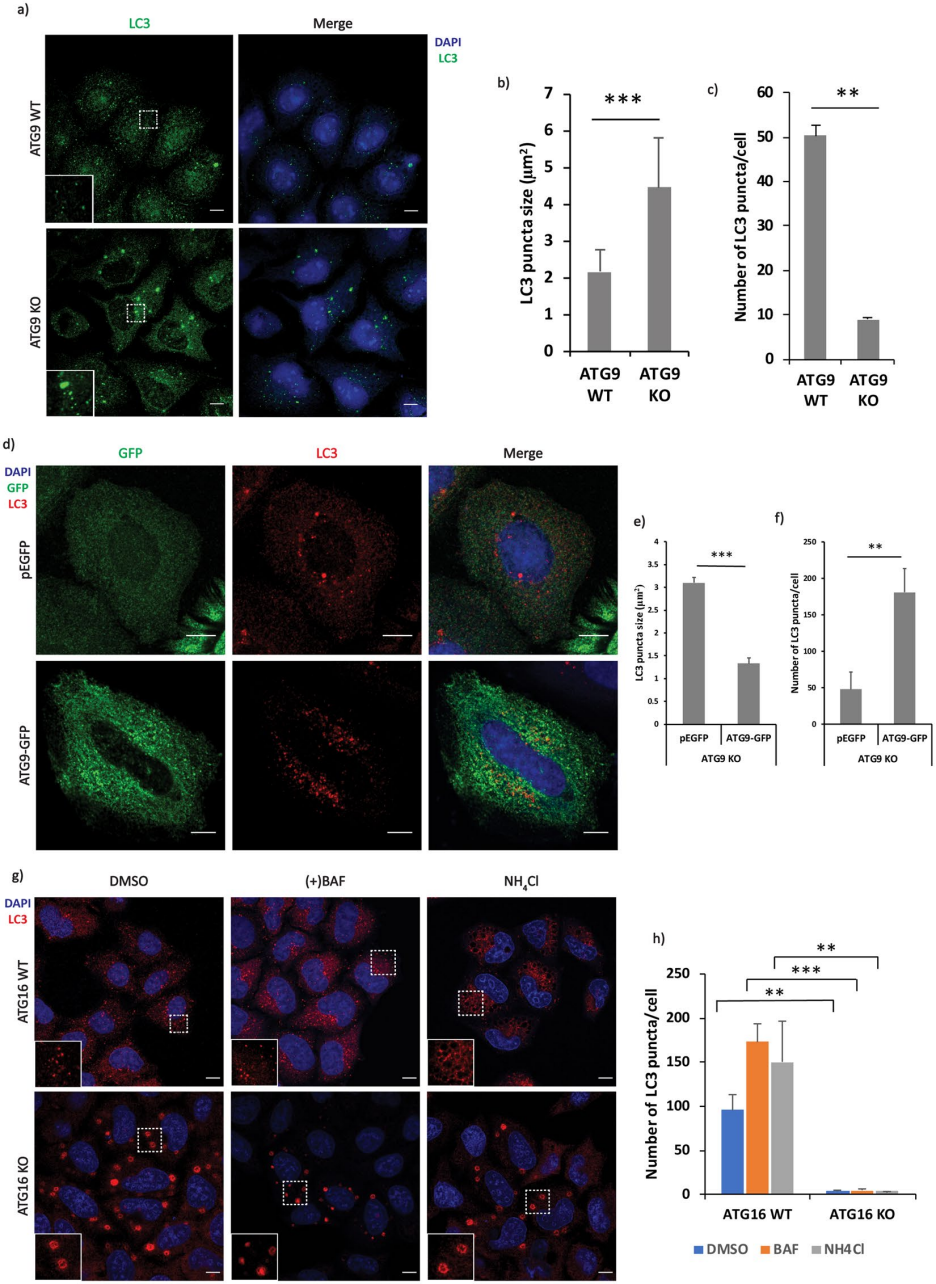
(**a**) Representative immunofluorescence images showing LC3 staining in ATG9 control and knockout cells. (**b**,**c**) Quantification of the LC3 puncta average size and average number from the images in (**a**) performed using ImageJ quantification tool (**p < 0.01, ***p < 0.001). Quantification performed from 3 experiments with >25 cells quantified for each condition. Error bars represent standard deviation (SD). (**d**) Representative images showing the rescue of the phenotype observed in ATG9-knockout cells using ATG9A-GFP construct. (**e**,**f**) Quantification of the LC3 puncta average size and average number from the images in (**d**) performed using ImageJ quantification tool (**p < 0.01, ***p < 0.001). Quantification performed from 3 experiments with >25 cells quantified for each condition. Error bars represent standard deviation (SD). (**g**) Representative immunofluorescence images showing LC3 staining in ATG16L1 control and knockout cells. ATG16 control and knockout cells were fixed with methanol and labelled for endogenous LC3. (**h**) Quantification of the average number of LC3 puncta as observed in the images shown in panel (g) performed using ImageJ quantification tool (**p < 0.01, ***p < 0.001). Quantification performed from 2 experiments with >25 cells quantified for each condition. Error bars represent standard deviation (SD). Scale bars represent a distance of 10 µm.

While characterising ATG9 CRISPR knockout HeLa cells (Supp. Fig. 1a), we found that LC3-II levels were slightly reduced in these cells in normal conditions but were dramatically reduced compared to wild-type cells when cells were treated with bafilomycin A1 (a lysosomal inhibitor that blocks LC3-II degradation and thus allows one to infer LC3-II formation rates; Supp. Fig. 1b,c). This was associated with a clear increase in LC3-I in ATG9 knockout cells (Supp. Fig. 1b,d). These phenotypes were rescued when the ATG9 null cells were transfected with ATG9A-GFP (Supp. Fig. 1e,f). To confirm this observation, we used LC3 immunocytochemistry (Fig. 1a). Interestingly, the ATG9 knockout cells had fewer, but much larger LC3-positive structures, compared to control cells (Fig. 1b,c). This phenotype was also rescued by transfection of the ATG9-null cells with ATG9A-GFP (Fig. 1d–f and Supp. Fig. 1 g).

We next studied ATG16L1 knockout HeLa cells^13^ that have no LC3-II (Supp. Fig. 1h), as expected^13^. Surprisingly, these cells also had large LC3-positive structures, compared to control cells (Fig. 1g,h), a phenomenon seen with different antibodies and fixation protocols (Supp. Fig. 1i). LC3-positive structures become more prominent in control cells after treatment with lysosomal pH-modulating autophagy inhibitors that compromise autophagosome degradation, like bafilomycin A1 and ammonium chloride. However, lysosomal alkalinisation did not alter the LC3-positive structures in the ATG16L1-null cells (Fig. 1g,h).

**Figure 2:**
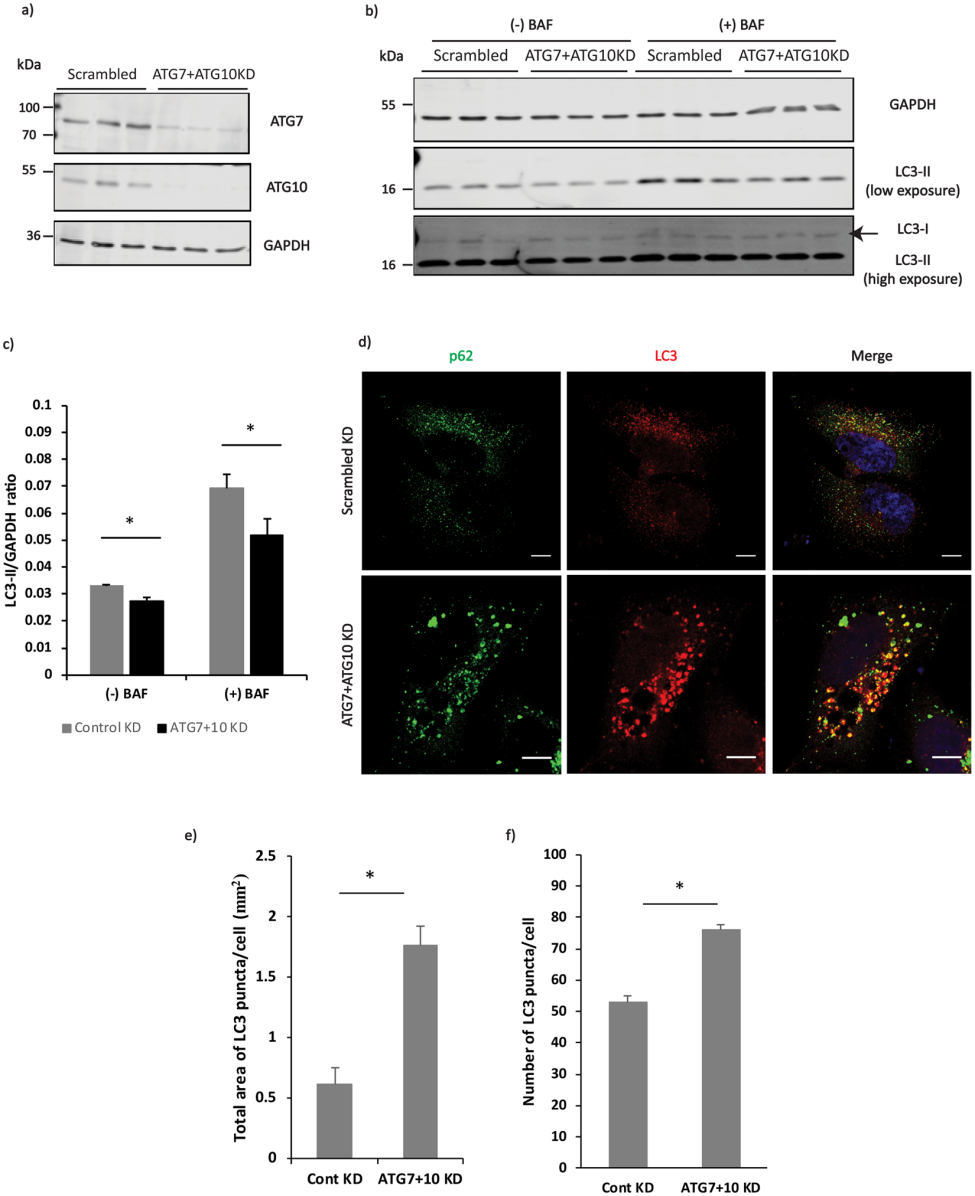
(**a**) Representative western blot showing the efficiency of ATG7 and ATG10 knockdown. (**b**) Representative western blot showing the decrease in LC3-II levels in cells upon ATG7 and ATG10 knockdown. (**c**) Quantification of the LC3-II levels with respect to levels of GAPDH upon depletion of ATG7 + 10 in HeLa cells. The statistical analysis was performed using one-tailed paired t-test. Error bars represent standard error of the mean (SEM). (**d**) Representative immunofluorescence images showing the morphology of LC3-positive structures under control and ATG7 + 10 knockdown conditions in HeLa cells. (**e**,**f**) Quantification of the LC3-positive structures’ average total area and number under control and ATG7 + 10 knockdown conditions using ImageJ quantification tool (*p < 0.05). Quantification performed from 3 experiments with >20 cells quantified for each condition. Error bars represent standard deviation (SD). Scale bars represent a distance of 10 µm. Full-length blots/gels are presented at the end of the Supplementary File.

To test whether the above observation could be replicated under conditions of acute autophagy knockdown in WT HeLa cells, we depleted ATG7 and ATG10 using siRNA-mediated knockdown (Fig. 2a). As expected, the LC3-II levels decreased upon ATG7 and ATG10 knockdown in the absence and presence of bafilomycin A1 (Fig. 2b,c). However, the total number of LC3-positive structures and the total area of the LC3-positive structures increased after siRNA-mediated depletion of ATG7 and ATG10 (Fig. 2d–f). To ensure that the observed effect of ATG7 and ATG10 knockdown was not artefactual, we performed a rescue of the phenotype using overexpression of ATG7 and ATG10-FLAG constructs. Our results showed that the knockdown phenotype was rescued upon overexpression of ATG7 and ATG10 constructs (Supp Fig. 2a–f).

### Non-conjugatable forms of LC3 form distinct LC3-I positive structures in autophagy-deficient cells

**Figure 3:**
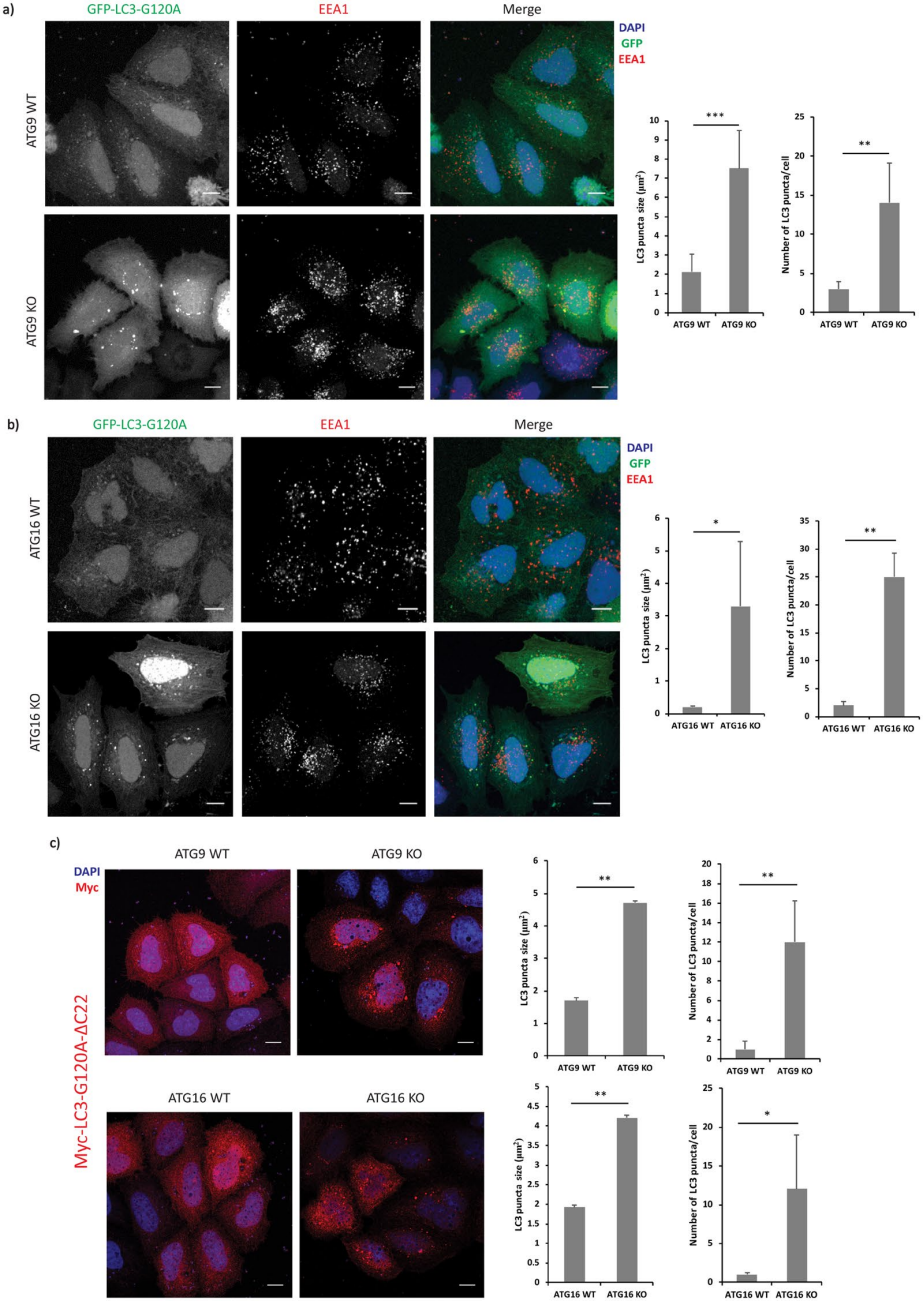
(**a**) Representative immunofluorescence images showing GFP-LC3G120A and early endosomal marker, EEA1 staining in ATG9 control and knockout cells. The graphs represent the quantification of LC3 puncta average size and average number in ATG9 control and knockout cells overexpressing GFP-LC3G120A using ImageJ quantification tool (**p < 0.01, ***p < 0.001). Quantification performed from 3 experiments with >25 cells quantified for each condition. Error bars represent standard deviation (SD). (**b**) Representative immunofluorescence images showing GFP-LC3G120A and early endosomal marker, EEA1, staining in ATG16L1 control and knockout cells. The graphs represent the quantification of LC3 puncta average size and average number in ATG16L1 control and knockout cells overexpressing GFP-LC3G120A using ImageJ quantification tool (*p < 0.05, **p < 0.01). Quantification performed from 3 experiments with >25 cells quantified for each condition. Error bars represent standard deviation (SD). (**c**) Representative immunofluorescence images showing Myc-LC3G120A-ΔC22 staining in ATG9, ATG16L1 control and knockout cells. The graphs represent the quantification of LC3 puncta average size and average number in these cells overexpressing Myc-LC3G120A-∆C22, using ImageJ quantification tool (*p < 0.05, **p < 0.01). Quantification performed from 2 experiments with >25 cells quantified for each condition. Error bars represent standard deviation (SD). Scale bars represent a distance of 10 µm.

As ATG16L1-null cells form LC3-positive structures, we confirmed whether LC3-I is capable of forming distinct structures in cells using non-conjugatable forms of LC3 tagged with GFP at the N-terminus of the protein. The conjugation of LC3-I to phosphatidylethanolamine can be abolished by mutating glycine 120 to alanine (LC3-G120A) to abrogate the ubiquitin-like reaction^14,15^. LC3 with this mutation can exist as pro-LC3 and LC3-I forms in the cell. However, LC3-G120A-ΔC22, where the glycine 120 to alanine mutation is combined with deletion of 22 amino acids located beyond the C-terminal alanine 120 residue (mimicking the ATG4 reaction), mimics LC3-I^15^. Both of these mutants formed prominent structures in ATG9 null or ATG16L1 null cells (Fig. 3a–c and Supp. Fig. 3a), similar to wild-type GFP-LC3. Furthermore, overexpression of non-conjugatable mutant forms of other LC3-family members such as GFP-GABARAP-GA and GFP-GABARAP-L1-GA in ATG16L1 KO cells resulted in the formation of structures similar to the ones described above (Supp. Fig. 3b,c). These findings demonstrate the formation of LC3-positive puncta in autophagy-impaired cells, even under conditions where the process of LC3-lipidation is completely abrogated, and other members of LC3-family behave in a similar fashion under autophagy-impaired conditions.

### The LC3-I positive structures are associated with p62 aggregates and are ubiquitin-positive

**Figure 4:**
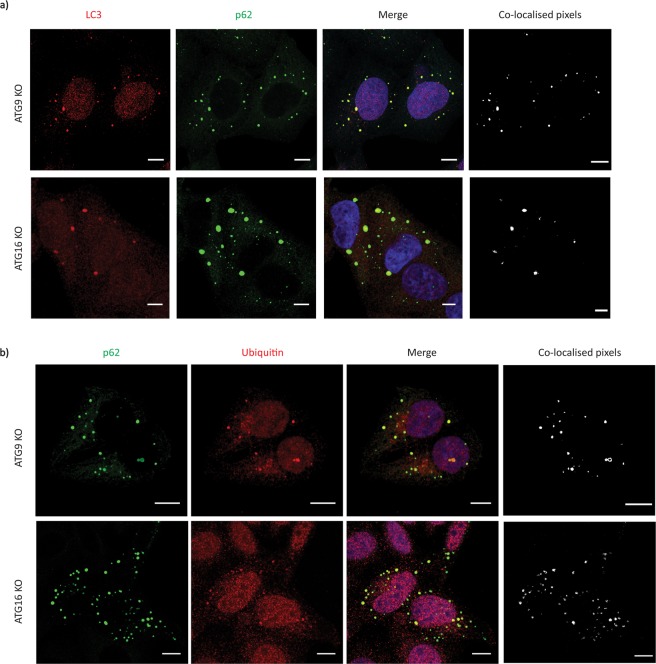
(**a**) Representative immunofluorescence images showing the co-localization of LC3-positive structures in autophagy-deficient ATG9 and ATG16L1 knockout cells with p62 using co-localisation profile generation. The co-localisation pixels for the image were identified, and a profile was generated using an unsupervised ImageJ plugin algorithm called colocalization, which was developed by Pierre Bourdoncle (Institut Jacques Monod, Service Imagerie, Paris; 2003–2004). (**b**) Representative immunofluorescence images showing the co-localization of p62 structures in autophagy-deficient ATG9 and ATG16L1 knockout cells with ubiquitin using co-localisation profile generation. The co-localisation pixels for the image were identified, and a profile was generated using an unsupervised ImageJ plugin algorithm called colocalization, which was developed by Pierre Bourdoncle (Institut Jacques Monod, Service Imagerie, Paris; 2003–2004). Scale bar represents a distance of 10 µm.

**Figure 5:**
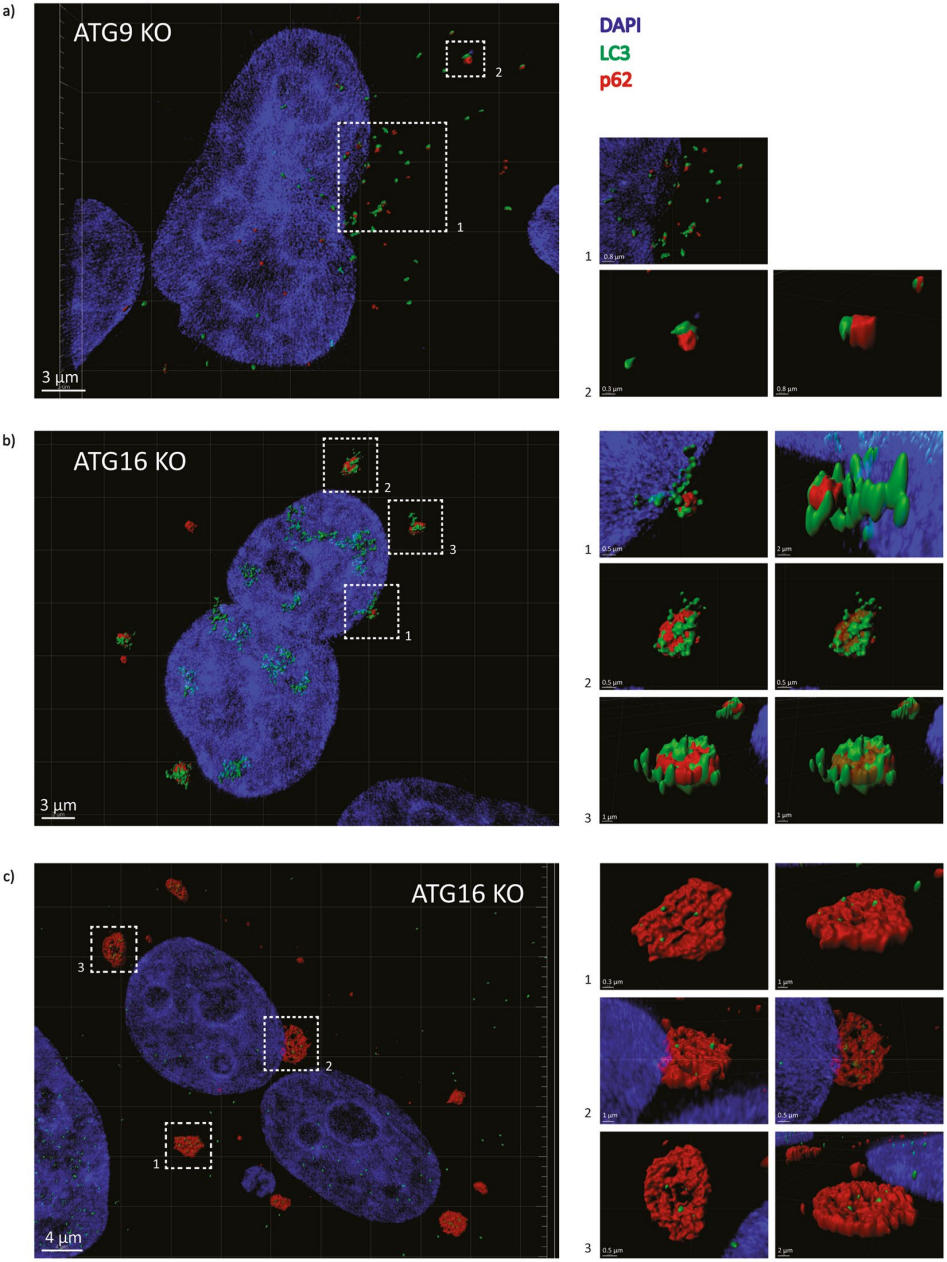
(**a**) Representative super-resolution immunofluorescence images showing the co-localization of LC3-positive structures with p62 in ATG9 knockout cells. (**b**) Representative super-resolution immunofluorescence images showing the co-localization of LC3-positive structures with p62 aggregates in ATG16L1 knockout cells. (**c**) Representative super-resolution immunofluorescence images showing the co-localization of LC3-positive structures with larger p62 aggregates in ATG16L1 knockout cells. Scale bars have been labelled individually for each magnification.

While these LC3-positive structures in ATG9-null and ATG16L1-null cells did not co-localise with a wide variety of endosomal (Fig. 3a,b, Supp. Fig. 4) and non-endosomal markers (Supp. Fig. 4), they appeared to co-localise perfectly with p62 (Fig. 4a and Supp. Fig. 4). Importantly, these p62 structures appeared to be ubiquitinated (Fig. 4b), suggesting that they represent p62 aggregates forming due to autophagy impairment^6^. Interestingly, since a recent study suggests that increased levels of LC3 inhibit the growth of p62 aggregates^16^, we therefore decided to further investigate the association of LC3 with p62. Super-Resolution Structured Illumination microscopy (SR-SIM) showed a clear association between LC3 and p62 structures (Fig. 5a–c). These p62 structures were much smaller in size in the ATG9-knockout cells compared to ATG16L1-knockout cells, which might be due to presence of some residual autophagy in ATG9-knockout cells. Moreover, some ATG16L1-knockout cells had extremely large p62 structures associated with little LC3. However, the number of these cells in the population was very low (less than ~1% of the total population) (Fig. 5c). Our impression was that smaller p62 structures appeared to have more LC3, compared to larger ones (Fig. 5b,c). These findings suggest that the LC3- and p62-positive structures present in autophagy-null cells may be aggregates.

**Figure 6:**
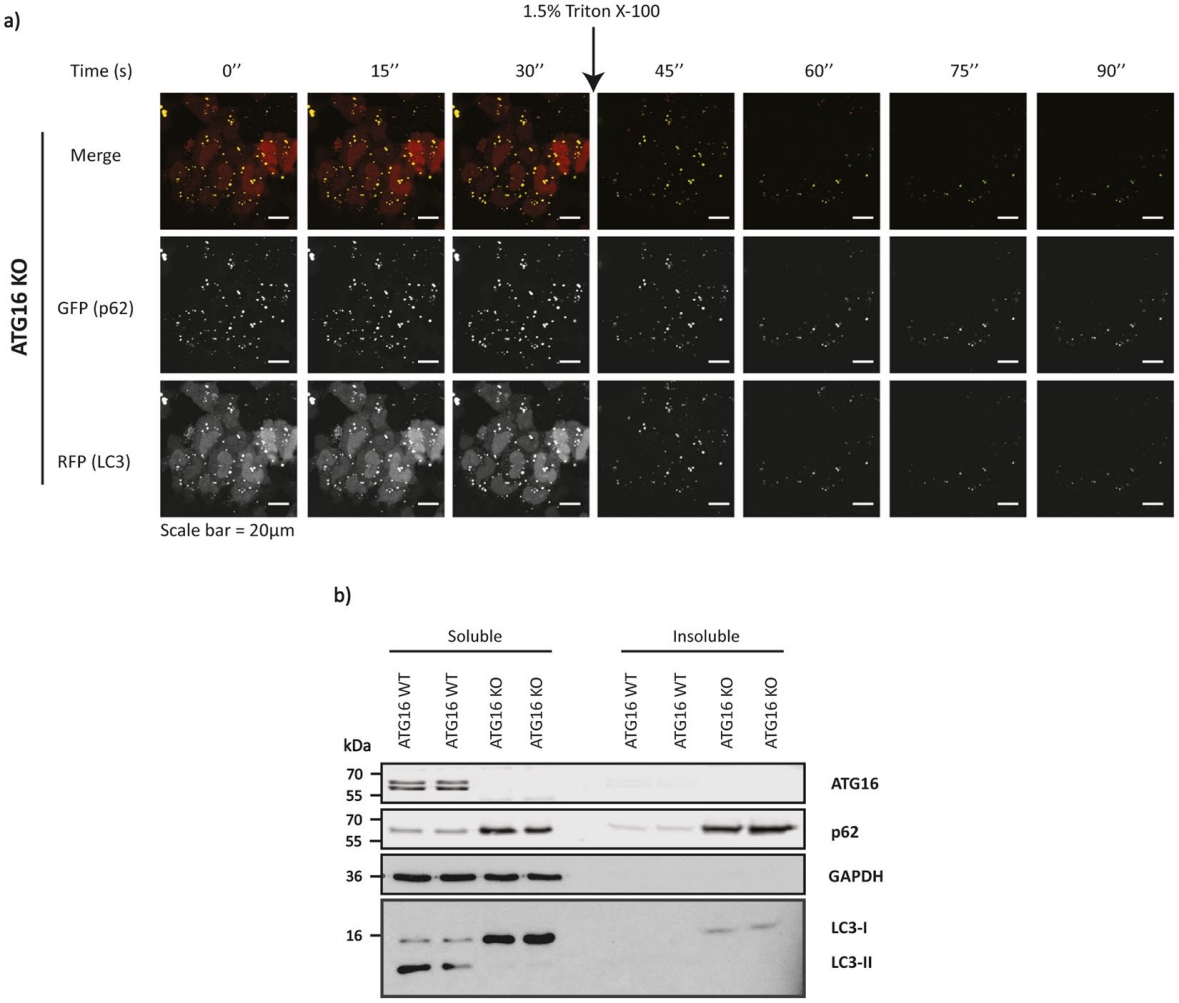
(**a**) Representative series of images depicting the live-cell imaging of cells during the detergent extraction experiment using 1.5% Triton X-100. ATG16L1-knockout cells expressing p62-pEGFP and RFP-LC3 were subjected to rapid detergent extraction by addition of 1.5% Triton X-100. The cells were imaged in a time series every 15 seconds for 1½ minutes. (**b**) Representative western blot showing the presence of LC3-I as well as p62 in the insoluble fraction of ATG16L1-knockout cells. The blot also shows the lack of ATG16L1 expression in ATG16L1-CRISPR knockout cells and a corresponding increase in the p62 levels in these cells. Please refer to Supplementary Information File for full-length blots. Scale bars represent a distance of 20 µm.

To confirm that the p62 structures in autophagy-impaired cells are indeed aggregates, as they co-localised with ubiquitin, we performed a rapid detergent extraction of the membranes in ATG16L1-knockout cells transfected with p62-pEGFP and RFP-LC3 (Fig. 6a and Supp. Fig. 5a,b). While the extraction results in the rapid loss of the RFP-LC3 signal within few seconds after addition of 1.5% Triton X-100, a significant fraction of RFP-LC3 remains associated with p62-pEGFP positive structures (Fig. 6a, Supp. Fig. 5a,b). Moreover, upon separation of the cell lysates into soluble and insoluble fractions, LC3-I as well as p62 were observed to be associated with the insoluble fraction in ATG16L1-knockout cells (Fig. 6b). These findings demonstrate that LC3-I is sequestrated by p62 aggregates in autophagy-impaired cells.

### p62 acts as a platform for LC3-I accumulation and is necessary for the formation of LC3-I positive structures in autophagy-deficient cells

We then tried to investigate whether the binding of LC3 and p62 is required for the formation of these LC3-positive structures observed in autophagy-impaired cells. We first excluded the possibility that our results are due to increased binding between LC3 and p62 in ATG9- and ATG16L1-knockout cells by overexpressing GFP-LC3 and performing GFP-trap assays to pull-down GFP-LC3 and probing for p62 levels. We observed no changes in the binding of LC3 to p62 in ATG9-knockout cells, whereas we found impaired binding in ATG16L1-knockout cells (Supp. Fig. 5c,d). The decrease in the LC3/p62 interaction observed in ATG16L1-knockout cells could be due to increased p62 aggregation and precipitation in the insoluble fraction (Supp. Fig. 5c,d). We then expressed mutant forms of LC3, which are defective for p62 binding^17,18^. Our results showed that the average size and average number of LC3-positive structures in ATG9- and ATG16L1-knockout cells expressing p62-binding defective mutant(s) was significantly decreased, compared to wildtype GFP-LC3 construct overexpression (Supp. Fig. 6a–d), demonstrating that LC3 and p62 binding is important for the formation of the aberrant LC3-positive structures observed in autophagy-impaired cells.

**Figure 7:**
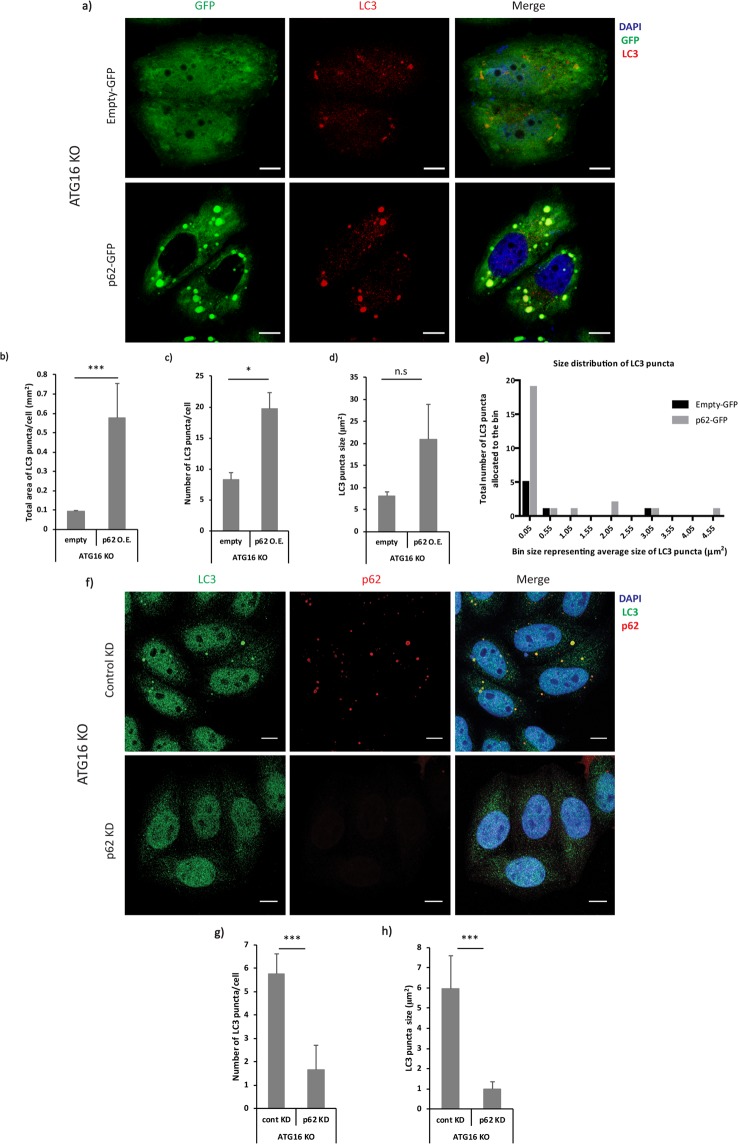
(**a**) Representative immunofluorescence images showing the morphology of LC3-I positive structures under control and p62-GFP overexpression conditions in ATG16 knockout cells. (**b**–**d**) Quantification of the LC3-positive structures’ total area, average number and average size under control and p62 overexpression conditions using ImageJ quantification tool (*p < 0.05, ***p < 0.001, n.s = non-significant). Quantification performed from 3 experiments with >25 cells quantified for each condition. Error bars represent standard deviation (SD). Scale bars represent a distance of 10 µm. (**e**) A histogram representing the frequency distribution of the estimated sizes of LC3 puncta from representative ATG16L1-knockout cells expressing either empty-GFP or p62-GFP. The data was sorted into bin-size and bin-width of 0.50 µm^2^ and 0.05 µm^2^ respectively and a frequency distribution histogram was plotted using GraphPad Prism (version 5.01). (**f**) Representative immunofluorescence images showing the morphology of LC3-I positive structures under control and p62 knockdown conditions in ATG16L1 knockout cells. (**g–h**) Quantification of the LC3-positive structures’ average number and size under control and p62 knockdown conditions using ImageJ quantification tool (**p < 0.01, ***p < 0.001). Quantification performed from 3 experiments with >25 cells quantified for each condition. Error bars represent standard deviation (SD).

We then tested whether increased p62 levels were sufficient to cause the formation of prominent LC3-positive structures in autophagy-deficient cells by overexpressing a GFP-tagged version of p62 in ATG16L1 wildtype and knockout cells. The number and area of the LC3-positive structures increased remarkably upon p62 overexpression in the knockout but not in wildtype cells (Fig. 7a–c and Supp. Fig. 7a). The quantification of the size of these LC3-I positive structures was not statistically significant in p62-GFP overexpressed ATG16L1 knockout cells compared to control, due to formation of a high number of small p62 aggregates upon p62-GFP overexpression, which resulted in a large variance in size (Fig. 7d). Consistently, this difference could be clearly seen upon sorting the estimated sizes of LC3 puncta from representative ATG16L1-knockout cells overexpressing either empty-GFP or p62-GFP into equally-sized bins and depicting the frequency distribution in the form of a histogram (Fig. 7e). Consistent with our previous observations, we saw that overexpression of a p62 mutant defective for LC3-binding (via a mutation in LIR-domain)^19^ appeared to have a trend towards a decrease in the average size of LC3-positive structures in ATG16L1-knockout cells (Supp. Fig. 7b–d). Importantly, siRNA-mediated depletion of p62 (Supp. Fig. 8a,b) caused a clear reduction in the formation of LC3-positive structures in the autophagy-impaired cells, as measured by the average number and size of these LC3-I positive structures in ATG16L1-knockout cells, as well as in ATG9-knockout (Fig. 7f–h and Supp. Fig. 8c–e). Interestingly, siRNA-mediated depletion of p62 in ATG16L1-knockout cells also caused a significant reduction in LC3-I levels compared to control, suggesting that p62 impairs the degradation of LC3-I in autophagy-impaired cells (Supp. Fig. 8f,g). These experiments suggest that p62 acts as a platform for the formation of LC3-positive structures via its association with LC3-I in cells.

While larger LC3-positive structures have been seen by ourselves in autophagy-deficient HeLa cells, we also tested if this occurred in HepG2 cells. Our data showed that ATG7 + 10 knockdown decreased the number and size of LC3 structures (Supplementary Fig. 9). Thus, the phenomenon we have described may depend on cell-type and/or the extent to which autophagy is compromised.

## Discussion

LC3 immunocytochemistry/immunohistochemistry/immunofluorescence is one of the most commonly used assays for measuring autophagosome number^8^. The technique is based on the principle that LC3-II is membrane-associated and thus appears as distinct puncta in the cell and can be easily visualised and hence quantified^1^. Since LC3-II is regarded as a specific marker for autophagosomes, this assay can measure the number, size or area of the autophagosomes.

**Figure 8:**
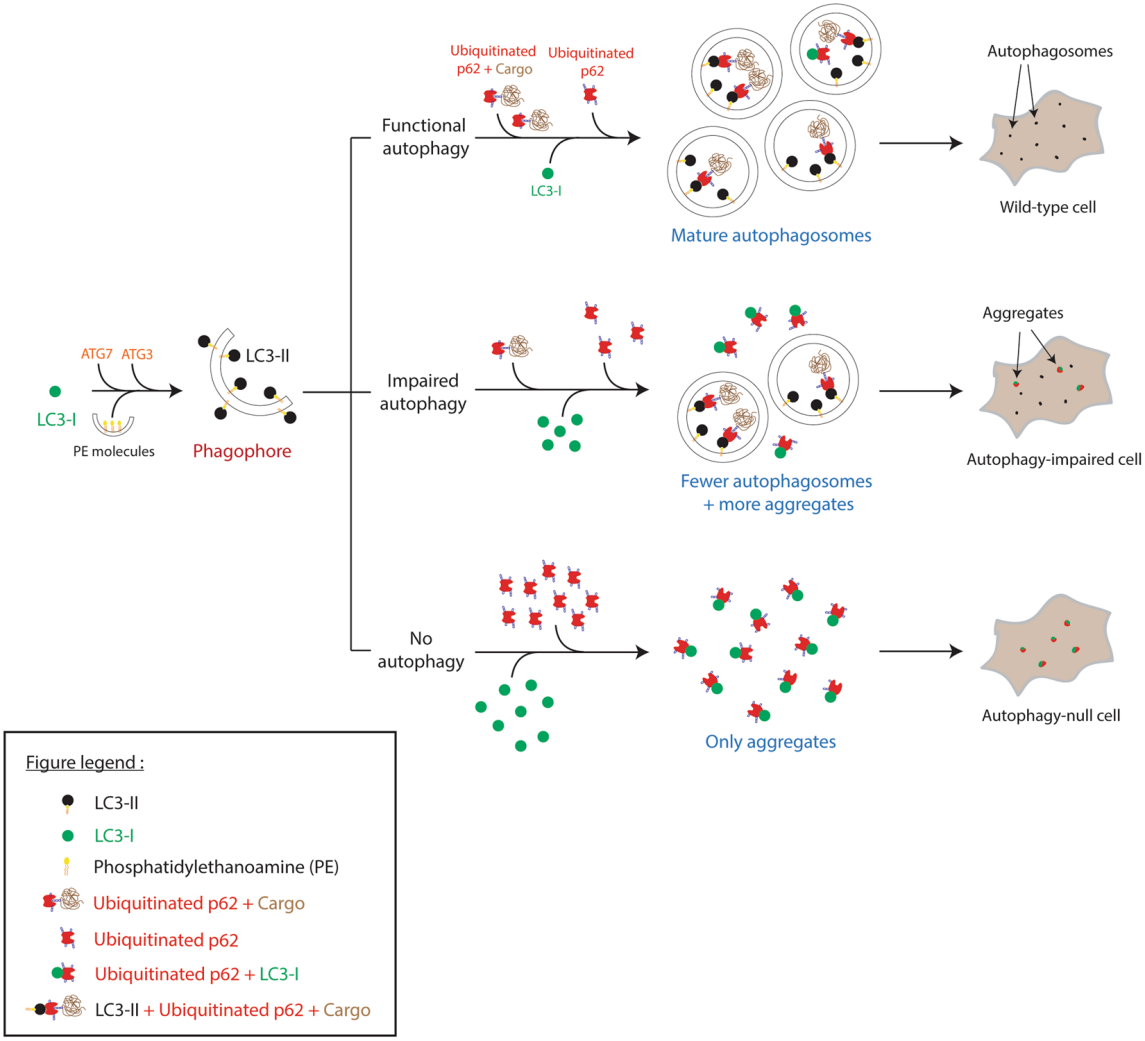
A schematic describing the process of LC3-I molecule sequestration by the p62 aggregates in autophagy-impaired and autophagy-null cells. The process of autophagy involves the lipidation of the cytosolic form of LC3 viz. LC3-I on to phosphotidylethanolamine (located on the growing phagophore) to form LC3-II, which is relatively specifically located on completed autophagosomes. The visualisation of LC3-II puncta using immunocytochemistry techniques is therefore considered to be a gold-standard assay to assess the autophagosome number in cells. Under conditions where autophagy is fully functional, LC3-I gets lipidated to form LC3-II, which results in the recruitment of ubiquitinated p62 attached to the cargo molecules destined for degradation. The p62-cargo complex is then engulfed into the growing autophagosomes and delivered to lysosomes for degradation. The LC3-II molecules then get delipidated/degraded to replenish the pool of cytosolic LC3-I in these cells. However, under conditions where LC3-I accumulates in cells as a result of autophagic impairment or complete abrogation, p62 aggregates (formed as a result of dysfunctional autophagy) sequester the excess LC3-I molecules forming structures that resemble autophagosomes. While these structures show an increased size compared to conventional autophagosomes, their presence might mislead an untrained eye, potentially resulting in confounding results. Please refer to the figure key for description of various symbols/structures.

Our findings in this study, consistent with previous observations^10–12^, suggest major caveats with this assay at least in some commonly used cell types. In the absence of LC3-II formation (ATG16L1-knockout cells), or when LC3-II formation is impaired (ATG9-knockout cells), endogenous LC3-I (and probably LC3-II) can form puncta with p62, which accumulate when autophagy is impaired^6,9^. This phenomenon occurs even with partial autophagy compromise after short-term knockdown of core autophagy genes. This can result in prominent LC3-positive structures, which may be confused with autophagosomes (Fig. 8). In some cases, one can see an increase of LC3-positive puncta when autophagosome formation is impaired, which was completely contrary to our expectations. This may be even more of an issue in *in vivo* experiments and has been shown to occur in ATG7-knockout mice^9^. Our data also suggest that one can get artefactual results if p62 expression levels are increased, or if p62 aggregation occurs when autophagy is perturbed^6^. It is also possible that the appearance of such LC3- and p62-positive aggregates may mirror a counteracting cellular pathway engaged in situations when autophagy is inhibited or impaired. For instance, LC3, which is also known to function in a form of phagocytosis (LC3-associated phagocytosis (LAP)^20^), may become functionally neutralised through p62 sequestration to avoid an imbalance in LAP under autophagy-impaired conditions. This could be a potential cellular response towards the stress imposed due to non-functional autophagy.

Also, while we only describe the involvement of p62 in this process, it is possible that some other autophagy receptors might behave in a similar way under autophagy deficiency or if their total levels are increased. Nevertheless, p62 seems both necessary and sufficient for the accumulation of endogenous (this study) and overexpressed (this study and^12^) LC3-I positive structures in autophagy-deficient conditions. While the appearance and number of LC3-positive structures in the presence of impaired autophagy is a concern, the extent to which this occurs and the numbers of these structures versus those seen in control cells may depend on the extent and period of autophagy inhibition and may be cell-type dependent. Importantly, the sequestration of LC3-I and likely LC3-II by p62 aggregates may also confound some other autophagy assays based on LC3 or LC3-family members such as GABARAP and GABARAP-L1.

## Materials and Methods

### Materials

The antibodies used in this study are: mouse anti-LC3B clone 5F10 (0231-100, NanoTools), rabbit anti-LC3B (EPR18709, Abcam), rabbit anti-LC3B for western blotting (NB100-2220, Novus biologicals) [Please note that the antibodies used against LC3 in this study specifically recognise only LC3B], mouse anti-p62 (610832/3, BD Biosciences), rabbit anti-p62 (PM045, MBL), mouse anti-GFP (A6455, Invitrogen), rabbit anti-RAB11 (715300, Invitrogen), mouse anti-EEA1 (ab70521, Abcam), rabbit anti-ATG9A (ab108338, Abcam), mouse anti-tubulin (T9026, Sigma), rabbit anti-ATG7 (ab52472, Abcam), rabbit anti-ATG10 (ab124711, Abcam), rabbit anti-ATG16L1 (D6D5, AB_10950320, Cell Signaling), rabbit anti-ATG16L1 (PM040, MBL), mouse anti-GAPDH (ab2107448, Abcam) and all the Alexa-conjugated secondary antibodies were purchased from Invitrogen. The pEGFP-LC3, pEGFP-LC3-G120A and RFP-LC3 constructs were kind gifts from Dr. T. Yoshimori (Osaka University, Japan). ATG9A-pEGFP was a kind gift from Dr Y. Takahashi (Penn State College of Medicine, USA), C1-mCherry was from Clontech (632524), mCherry-p62 and mCherry-p62 LIR mutant (DDDW335-338AAAA) was a kind gift from Dr. Sascha Martens (University of Vienna, Austria), p62-pEGFP was a kind gift from Dr. Terje Johansen (Artic University, Norway), while Myc-LC3-G120A-ΔC22 was a gift from Dr. T. Yoshimori (Addgene plasmid # 45449). The pSpCas9(BB)-2A-Puro (PX459) V2.0 was a gift from Dr. F. Zhang (Addgene plasmid # 62988). pCMV-ATG7 was a kind gift from Dr. Isei Tanida. EGFP-LC3K51A and EGFP-LC3F52A were created by site mutagenesis using the Q5 Site-Directed Mutagenesis kit (NEB E0554S) and pEGFP-LC3 as a template according to the manufacturer’s instructions. Primers used for the K51A mutation: 5′-ccgtcctggacaagaccgcgttccttgtacctgatc-3′ and for the F52A mutation: 5′-gtcctggacaagaccaaggcccttgtacctgatcacgt-3′. For creating the GFP-GABARAP-GA and GFP-GABARAPL1-GA we first generated GFP-GABARAP and GFP-GABARAPL1 by cloning the respective coding sequences (ordered from GeneScript) onto the pEGFP-C1 backbone (Clontech). Primer sequences used for GFP-GABARAP: 5′-CCCGAATTCCATGAAGTTCGTGTACAAAGA-3′ and 5′-CCCGGAT CCTCACAGACCGTAGACACTTTC-3′, for GFP-GABARAPL1: 5′-CCCGAATTCC ATGAAGTTCCAGTACAAGGA-3′ and 5′-CCCGGATCCTCATTTCCCATAGACAC TCTC-3′. The lipidation deficient mutants were made by introducing G116A (for GABARAP) and G142A (for GABARAPL1) mutations using the Q5 Site-Directed Mutagenesis kit (NEB E0554S) according to the manufacturer’s instructions. For GFP-GABARAP-G116A primers used were: 5′-AGTGTCTACGcTCTGTGAGGATC-3′ and 5′-TTCGTCACTGTAGG CAATG-3′. For GFP-GABARAPL1-G142A primers used were: 5′-CTTACATATGcCAGTGTAAGGC-3′ and 5′-AGGATCCGGATAAATAAC-3′.

### Cell culture

HeLa cells were cultured in Dulbecco’s Modified Eagle Medium (DMEM) supplemented with 10% v/v fetal bovine serum, 100 U/ml penicillin-streptomycin and 2 mM L-glutamine. HepG2 cells were maintained in RPMI 1640 medium supplemented with 10% v/v fetal bovine serum, 100 U/ml penicillin-streptomycin, all obtained from Sigma, UK. The cell lines were maintained by passaging the cells, using Trypsin-EDTA solution (Sigma), after they are sub-confluent to around 75–90% in T75 (75 cm^2^ area) flasks (Falcon). The cell line used for this study was HeLa (Human cervical cancer cells) cell line. In experiments where we needed to block autophagic flux, cells were treated with 400 nM of bafilomycin A1 for 4 hours at 37 °C.

### Transfection

The HeLa cells were transfected with the purified plasmid using the Mirus TransIT-2020 transfection reagent. The transfection was performed using optiMEM medium, using the manufacturer’s protocol. Briefly, a mixture of 100 μl of optiMEM with 1 μg of DNA was prepared and incubated at room temperature for 5 minutes. Another mixture containing 100 μl optiMEM with 5 μl of Mirus was prepared and incubated for 5 minutes. Both of the solutions were then mixed together and incubated for approximately 20–25 minutes. The total volume was then added to one well of a 6-well plate, already containing 1 ml of optiMEM. The amount of DNA generally used for transfection was 1 μg for a 6-well.

### siRNA-mediated knockdown

The cells for siRNA knockdown were transfected using lipofectamine 2000 transfection reagent. The transfection was performed using optiMEM medium, using the manufacturer’s protocol. Briefly, a mixture of 100 μl of optiMEM with 3 μl of 20 μM or 1 μl of 100 μM siRNA was prepared and incubated at room temperature for 5 minutes. Simultaneously, another mixture containing 100 μl optiMEM with 5 μl of Lipofectamine 2000 was prepared and incubated for 5 minutes. Both the solutions were then mixed together and incubated for approximately 20–25 minutes. The total volume was then added to one well of a 6-well plate, already containing 1 ml of optiMEM for 4 hours at 37 °C. After the incubation, the media was changed and replaced with full media. The cells were then harvested after 48 hours of incubation if a single knockdown (KD) was desired, as for the ATG7 and ATG10 KD in HeLa cells. For the ATG7 and ATG10 rescue experiments, a pcDNA.3 (empty vector) or ATG7 plus ATG10-FLAG were used to transfect cells 24hrs after the siRNA transfection. For double KD studies, we performed another round of siRNA transfection, following the same protocol as mentioned above, after the first round (for p62 KD studies). For siRNA knockdown in HepG2 cells, these were transfected using Lipofectamine RNAi max transfection reagent using 100 nM final siRNA and two rounds of siRNA transfection were performed to achieve an efficient knockdown. Cells were then split and seeded to respective plates or coverslips for further experiments. The scrambled siRNA (ON-TARGETplus Non-targeting Pool, D-001810-10), human ON-TARGETplus SQSTM1 (SMARTpool, L-010230-00-0005), human ON-TARGETplus ATG7 (SMARTpool, L-020112-00), human ON-TARGETplus ATG10 (SMARTpool, L-019426-01) were ordered from Dharmacon and used at a final concentration of 50–100 nM.

### GFP-trap assays

HeLa cells were transfected with EGFP-LC3 using Mirus TransIT-2020 transfection reagent and 24hrs later were lysed in lysis buffer (50 mM HEPES, 150 mM NaCl, 1% Triton X-100, 1.5 mM MgCl_2_, 5 mM EGTA) for 10 min on ice and pelleted for 10 min at 13,000 rpm. GFP-tagged proteins (EGFP or EGFP-LC3) were pulled down using GFP-TRAP beads (ChromoTek), according to the manufacturer’s protocol. Immunoprecipitates were eluted by boiling the samples in Laemmli buffer for 5 min. Proteins were resolved by SDS-PAGE.

### Immunofluorescence

The immunocytochemistry was performed on cells grown on 22 × 22 mm coverslips. HeLa cells were grown at a confluency of 70–80%, washed once with PBS and then fixed either using 4% w/v PFA for 5–7 minutes or cold-methanol for 3–5 minutes at 4 °C. Please note that since most of the antibodies used in the study worked only upon PFA fixation, the samples were generally fixed with 4% w/v PFA in PBS unless otherwise mentioned in the figure legend(s). PFA was discarded in accordance with the safety regulations and the cells were washed thrice with PBS. The cells were then permeabilised using 0.5% v/v Triton X-100 for 5–7 minutes and washed three times with PBS to remove any residual detergent. A solution containing 1% w/v BSA was then added onto the coverslips, as a blocking solution, to reduce the non-specific binding of the primary and secondary antibodies, and kept for 1 hour at room temperature. The blocking solution was then tapped off the coverslips and the primary antibodies at appropriate dilutions were added on the coverslips. The coverslips with primary antibodies were incubated at 4 °C for 16–20 hours in a moist and humid chamber. The coverslips were next washed three times with PBS and incubated with a solution containing secondary antibodies for 1 hour at room temperature. (Note that the primary and secondary antibody solutions were made in the blocking buffer.) The dilution of the secondary antibodies used for this study was 1:400 prepared in 1% w/v BSA solution. Finally, coverslips were washed twice with PBS and high purity sterile water and mounted on glass slides using Pro-Long gold anti-fade DAPI mounting medium (Invitrogen, US). The coverslips were then imaged using the Zeiss LSM880 or LSM780 confocal microscope using the 63x oil immersion objective.

### Live-cell imaging

ATG16L1-knockout cells were seeded on to the MatTek dishes (MatTek, Ashland MA USA). The following day, these cells were transfected with appropriate plasmid based on experimental requirements using Mirus Trans-IT 2020 transfection reagent. The transfection was performed using optiMEM medium, using the manufacturer’s protocol. A 2% Triton X-100 stock solution was then used to reach the designated concentration for detergent extraction of the plasma membrane. A final concentration of 1% Triton X-100 is capable of extracting the cytosolic and membrane bound. The experiment to confirm the presence of p62 aggregates in ATG16L1-knockout cells, however, involved detergent extraction using 1.5% Triton X-100 as the final concentration to subject the cells to harsher conditions of extraction. Imaging then was performed using the ‘Time series’ module on an incubated Zeiss AxioObserver Z1 microscope with a LSM780 confocal microscope using a 63 × 1.4 NA Plan Apochromat oil-immersion lens.

### Super-resolution microscopy

Samples were seeded onto Zeiss High precision No 1.5 170+ or −5 μm, 18 mm × 18 mm coverslips. Following staining, the samples were mounted in Pro-Long gold anti-fade DAPI mounting medium (Life Technologies) and left to harden for 3 days to reach a constant Refractive Index (RI) of 1.46. Samples were imaged using Structured Illumination on the Elyra PS1 (Carl Zeiss Microscopy). Following stage alignment, laser lines of 405, 488 and 561 nm were used to image a bead stack in order to correct for chromatic aberration using the channel alignment algorithm. Z-stacks were acquired at 5 phases and 5 rotations of the illumination grid and subsequently processed and aligned using the ZEN Black Elyra edition software (Carl Zeiss Microscopy).

### Extraction of soluble and insoluble fractions

The extraction of the soluble and insoluble fractions from ATG16L1-knockout cells was performed in RIPA buffer. Briefly, the cells were washed with 1X ice-cold PBS once and were scraped, lysed in 500 µl of RIPA buffer and collected in labelled tubes kept on ice. The composition of RIPA buffer was as follows –

**RIPA buffer**

50 mM Tris-HCl (pH 7.4)

150 mM NaCl

5 mM EDTA

1% Triton X-100

0.5% Sodium deoxycholate

0.1% SDS

At the time of use, RIPA buffer was supplemented with cOmplete protease inhibitor tablet (1 tablet per 50 ml) and phosphatase inhibitor cocktails 2 and 3 (1:100 each).

The lysates were then passed through a 25 G needle 10 times and then the solution was centrifuged at 14000 rpm for 15 minutes at 4 °C. After centrifugation, the supernatant was collected in a fresh Eppendorf tube and was labelled as the soluble fraction and was mixed with 300 µl of 2X Laemmli buffer and was boiled for 5 minutes. The pellet obtained after centrifugation consists of the insoluble fraction and was resuspended and washed with 500 µl of RIPA buffer. The resuspended solution was then centrifuged at 14000 rpm for 5 minutes at 4 °C. The supernatant was discarded and the pellet was resuspended in 100 µl of UREA + RIPA buffer. This was finally mixed with 100 µl of 2X Laemmli buffer and was boiled for 5 minutes. The composition of UREA + RIPA buffer consists of additional Urea at a final concentration of 2 M together with the components of RIPA buffer.

### Western blot

Cells cultured in 6-well plates were lysed and collected in 2X Laemmli buffer (4% w/v SDS, 20% v/v glycerol, 10% v/v 2-mercaptoethanol, 0.004% w/v bromophenol blue and 125 mM Tris HCl, pH 6.8). After the cells were lysed, 20–30 μl of the samples were loaded onto a 10-well, 12–15% SDS-PAGE and run at 100–120 V. A standard M.W. ladder was loaded along with the samples to keep a track of the movement of the proteins in the gel. The proteins were then transferred onto the activated PVDF at 100 V for 60 minutes. The membrane was removed on completion of the transfer and was soaked in 5% w/v skimmed milk to block non-specific binding sites, for 1 hour at room temperature. Next, a solution containing specific primary antibody (prepared in 5% w/v skimmed milk) at an appropriate dilution was added on to the membrane for overnight incubation at 4 °C. The membranes were then washed three times with PBST (Phosphate buffer saline +0.1% Tween-20) before the addition of secondary antibody. The secondary antibody, also prepared in 5% w/v skimmed milk, was incubated with the membrane for 1 hour at room temperature. The dilution of the secondary antibody was generally kept at 1:4000 for enhanced chemiluminescence (ECL) HRP-conjugated antibodies and 1:5000 for LICOR fluorophore-conjugated antibodies. The membranes were then washed again with PBST multiple times and developed using a mixture of equal volumes of developing solutions 1 and 2 for ECL or imaged directly for fluorescence signal detection using LICOR Image Studio software (LICOR, US).

### CRISPR knockout cell generation

The guide RNAs were designed for the generation of ATG9 knockout cells using the online tool developed by the Zhang Lab^21^. The top three single guide RNA (sgRNA) hits were modified manually to add BbsI restriction sites and these sgRNA sequences of desalted purity were ordered from Invitrogen. The sequence successfully used for ATG9 CRISPR knockout was:

**Table:** 

	**Sequences** **(5′ to 3′)**
**sgRNA**	CACCGCTGTTGGTGCACGTCGCCGA
	AAACTCGGCGACGTGCACCAACAGC

The highlighted nucleotides indicate the added overhangs. The sgRNA was then ligated in the psCas9-2A-puromycin backbone. The successfully ligated plasmids or empty Cas9 vector control were then transfected in HeLa cells and the expression was allowed for 24 hours. Transfected cells were then selected by adding puromycin to the cells at a final concentration of 2–4 µg/ml. Cells were allowed to grow until all the non-transfected cells were dead. The positive cells were then trypsinised and the cell number was assessed using Invitrogen Countess slides (10 µl homogenous cell suspension +10 µl Trypan blue). Based on the live cell population, 0.2 × 10^4^ cells were aspirated and serially diluted in a 96-well plate to obtain single cell colonies. The serial dilution was always 1:1 and the final volume in each well at the end of dilution was kept to 200 µl. The cell lysates were then loaded on SDS-PAGE, transferred to a membrane to check for the knock-out of ATG9.

### Quantification of data and Statistical analysis

The images were quantified using the default ‘Analyze particles’ plugin in ImageJ. Additionally, the co-localised pixels were identified, and a profile was generated using an unsupervised ImageJ plugin algorithm called ‘colocalization’, which was developed by Pierre Bourdoncle (Institut Jacques Monod, Service Imagerie, Paris; 2003–2004). The western blots were quantified using the LICOR Image Studio software (LICOR, US) and the values were processed, analysed and plotted using Excel program from the Microsoft Office suite.

The statistical significance levels for comparisons between two groups were estimated using one-tailed or two-tailed *t*-tests. For the experiment involving ATG7 and ATG10 KD, one-tailed *t*-test was used to assess the statistical significance of the data. For all the other experiments in this study, a two-tailed *t*-test was used to assess the statistical significance of the data. In this paper, the value of p < 0.05 was considered statistically significant and the conventions used to depict the results across this article are as follows: *p ≤ 0.05; **p ≤ 0.01; ***p ≤ 0.001 and Error bars represent standard deviations unless otherwise mentioned.

## Supplementary information

Supplementary Figures and legends and uncropped blots
