# Supplementary material for: LC3-positive structures are prominent in autophagy-deficient cells
Source: Sci Rep. 2019 Jul 12;9:10147. doi: 10.1038/s41598-019-46657-z (PMC6625982; doi:10.1038/s41598-019-46657-z)

LC3-positive structures are prominent in autophagy-deficient cells

Gautam Runwal[1], Eleanna Stamatakou[1,2], Farah Siddiqi[1,2], Claudia Puri[1,2], Ye Zhu[1] and David C. Rubinsztein[1,2]*

[1]*Department of Medical Genetics, Cambridge Institute for Medical Research;* [2]*UK Dementia Research Institute, The Keith Peters Building, Cambridge Biomedical Campus, Hills Road, Cambridge CB2 0XY, UK.*

* Lead Contact and Author for Correspondence: dcr1000@cam.ac.uk

Supplementary figure 1. a) Representative western blots showing the lack of ATG9 expression in ATG9 knockout cells. b) Representative western blots showing the LC3-II and LC3-I levels in ATG9 control and knockout cells. Please refer to Supplementary information file for full-length blots. c) Quantification of the intensities of the LC3-II bands, using LICOR-imaging software, observed in panel b) of this figure. The LC3-II values were then plotted against the values of the loading control, tubulin. The data is from the quantification of three experiments in triplicates and Error bars represent the standard error of the mean (SEM). d) Quantification of the intensities of LC3-I bands observed in panel b) of this figure. The LC3-I values were then plotted against the values of the loading control, tubulin (* = $p < 0.05$, ** = $p < 0.01$, *** = $p < 0.001$, n.s. = non-significant). The data is from the quantification of three experiments in triplicates and Error bars represent SEM. e) Representative western blot depicting the rescue of ATG9 knockout cells using ATG9A-GFP construct in the absence and presence of BAF. f) Quantification of the blot performed using LICOR-imaging software (* = $p < 0.05$, ** = $p < 0.01$, *** = $p < 0.001$, n.s. = non-significant). The data is from the quantification of three experiments in triplicates and Error bars represent SEM. g) Representative immunofluorescence image showing the expression of empty-GFP vector in ATG9 WT cells. h) Representative western blots showing the lack of ATG16L1 expression in ATG16L1 knockout cells. i) Representative immunofluorescence images showing LC3 staining using two different antibodies from different manufacturers. These antibodies work with specific fixation methods as shown in the figure. Scale bars represent a distance of 10μm. Full-length blots/gels are presented at the end of the Supplementary file.

Supplementary figure 2. a) Representative immunofluorescence images showing the rescue of ATG7 and ATG10 knockdown experiment by overexpression of ATG7 and FLAG-tagged ATG10 in HeLa cells. b), c) Quantification of the LC3 puncta average size and average number from the images in a) performed using ImageJ quantification tool (* = $p < 0.05$, ** = $p < 0.01$, *** = $p < 0.001$, n.s. = non-significant). d), e) Quantification of the p62 puncta average size and average number of the images in a) performed using ImageJ quantification tool (* = $p < 0.05$, ** = $p < 0.01$, *** = $p < 0.001$, n.s. = non-significant). Quantification performed from 3 experiments with >25 cells quantified for each condition. Error bars represent standard deviation (SD). Scale bars represent a distance of 10μm. f) Representative western blot showing the efficiency of the knockdown and rescue. Full-length blots/gels are presented at the end of the Supplementary file.

Supplementary figure 3. a) Representative immunofluorescence images showing the distribution of GFP-GABARAP-GA mutant in ATG16L1 control and knockout cells. b) Representative immunofluorescence images showing the co-localisation of GFP-GABARAP-GA and GFP-GABARAP-L1-GA lipidation defective mutants with p62 positive structures in ATG16L1-knockout cells. Scale bars represent a distance of 10μm.

Supplementary figure 4. Representative immunofluorescence images showing the co-localisation of LC3 puncta with various endosomal markers, syntaxin-17 and p62 in representative ATG9 knockout cells using co-localisation profile generation. The co-localisation pixels were identified, and a profile was generated using an unsupervised ImageJ plugin algorithm called colocalization, which was developed by Pierre Bourdoncle (Institut Jacques Monod, Service Imagerie, Paris; 2003-2004). The cells were labelled for endogenous LC3 which recognises both forms of LC3 viz. LC3-I and LC3-II together with antibodies against small molecule GTPase effectors which act as conventional markers for respective endosomal compartments such as RAB5 for early endosomes, RAB7 for late endosomes and RAB11 for recycling endosomes. The co-localisation between the LC3 puncta was also tested against the SNARE-protein STX17 and the common autophagy receptor, p62. The extent of co-localisation between the different marker pairs can be visualised using the grey pixels in the co-localisation pixels panel. Scale bars in the images represent a distance of 10μm.

Supplementary figure 5. a) Representative immunofluorescence images indicating the presence of p62-GFP and LC3-RFP association in ATG16L1-knockout cells at the initial and final time points of adding 1.5% Triton X-100. b) Representative immunofluorescence images of a time-series live-cell experiment to confirm the association of p62-GFP aggregates with LC3-RFP in ATG16L1-knockout cells. The frames represent images taken after every 15 seconds for 2½ minutes and the arrows indicate the addition of designated concentration of Triton X-100 to the cells. Scale bars represent a distance of 10μm unless stated otherwise in the figure. c) Representative western blot depicting the binding of GFP-LC3 to p62 using GFP-trap. Please refer to Supplementary information file for full-length blots. d) Quantification of the blot performed using LICOR-imaging software (* = $p < 0.05$, ** = $p < 0.01$). The data is from the quantification of three experiments in triplicates and Error bars represent SEM.

Supplementary figure 6. a) Representative immunofluorescence images depicting the GFP staining in ATG9-knockout cells expressing either GFP-LC3, GFP-LC3-F52A or GFP-LC3-K51A. b) Representative

immunofluorescence images depicting the GFP staining in ATG16L1-knockout cells expressing either GFP-LC3, GFP-LC3-F52A or GFP-LC3-K51A. c) Quantification of the LC3 puncta average size and average number in cells overexpressing either GFP-LC3, GFP-LC3F52A or GFP-LC3K51A in ATG9 control or ATG9 knockout cells performed using ImageJ quantification tool (* = $p < 0.05$, ** = $p < 0.01$, *** = $p < 0.001$, n.s. = non-significant). Quantification performed from 3 experiments with >25 cells quantified for each condition. Error bars represent standard deviation (SD). d) Quantification of the LC3 puncta average size and average number in cells overexpressing either GFP-LC3, GFP-LC3F52A or GFP-LC3K51A in ATG16L1 control or ATG16L1 knockout cells performed using ImageJ quantification tool (* = $p < 0.05$, ** = $p < 0.01$, *** = $p < 0.001$, n.s. = non-significant). Quantification performed from 3 experiments with >25 cells quantified for each condition. Error bars represent standard deviation (SD). Please note that cells with similar expression levels across the 3 variants of LC3 were imaged and quantified for this experiment. Scale bars represent a distance of 10μm.

Supplementary figure 7. a) Representative immunofluorescence images showing the overexpression of either p62-mCherry or p62-LIR-mCherry mutant in ATG16L1 knockout cells. b) Quantification of the LC3 puncta average size and average number in ATG16 control cells from the images in a) performed using ImageJ quantification tool (* = $p < 0.05$, ** = $p < 0.01$, *** = $p < 0.001$, n.s. = non-significant). Quantification performed from 3 experiments with >25 cells quantified for each condition. Error bars represent standard deviation (SD). d) Quantification of the LC3 puncta average size and average number in ATG16L1 knockout cells from the images in a) performed using ImageJ quantification tool (* = $p < 0.05$, ** = $p < 0.01$, *** = $p < 0.001$, n.s. = non-significant). Quantification performed from 3 experiments with >25 cells quantified for each condition. Error bars represent standard deviation (SD). Please note that cells with similar expression levels across the 3 variants of LC3 were imaged and quantified for this experiment. Scale bars represent a distance of 10μm.

Supplementary figure 8. a) Representative western blot showing the efficiency of p62 knockdown in ATG9 control and knockout cells. Please refer to Supplementary information file for full-length blots. b) Representative western blot showing the efficiency of p62 knockdown in ATG16L1 control and knockout cells. c) Representative immunofluorescence images showing the morphology of LC3-I positive structures under control and p62 knockdown conditions in ATG9 knockout cells. d), e) Quantification of the LC3-positive

structures' average number and size under control and p62 knockdown conditions using ImageJ quantification tool (* = $p < 0.05$, ** = $p < 0.01$, *** = $p < 0.001$, n.s. = non-significant). Quantification performed from 3 experiments with >25 cells quantified for each condition. Error bars represent standard deviation (SD). Scale bars represent a distance of 10µm. f) Representative western blot showing the effect of p62 knockdown in ATG16 control and knockout cells on LC3-I. g), h) Quantification of the blot performed using LICOR-imaging software (* = $p < 0.05$, ** = $p < 0.01$, *** = $p < 0.001$, n.s. = non-significant). The data is from the quantification of three experiments in triplicates and Error bars represent SEM. i) Representative western blot showing the effect of p62 knockdown in ATG9 control and knockout cells on LC3-I. Please refer to Supplementary information file for full-length blots. j) Quantification of the blot performed using LICOR-imaging software. The data is from the quantification of three experiments in triplicates and Error bars represent SEM. Full-length blots/gels are presented at the end of the Supplementary file.

Supplementary figure 9. a) Representative immunofluorescence images showing the LC3 and p62 staining in HepG2 cells upon ATG7 and ATG10 knockdown. b), c) Quantification of the LC3-positive structures' total size and average number in HepG2 cells using ImageJ quantification tool (*** = $p < 0.001$). Quantification performed from 3 experiments with >25 cells quantified for each condition. Error bars represent standard deviation (SD). Scale bars represent a distance of 10µm. d)- e) Representative western blot showing the efficiency of ATG7 and ATG10 knockdown, the decrease in LC3-II levels and the increase in p62 levels upon ATG7 and ATG10 knockdown. Full-length blots/gels are presented at the end of the Supplementary file.

Supplementary Figure 1

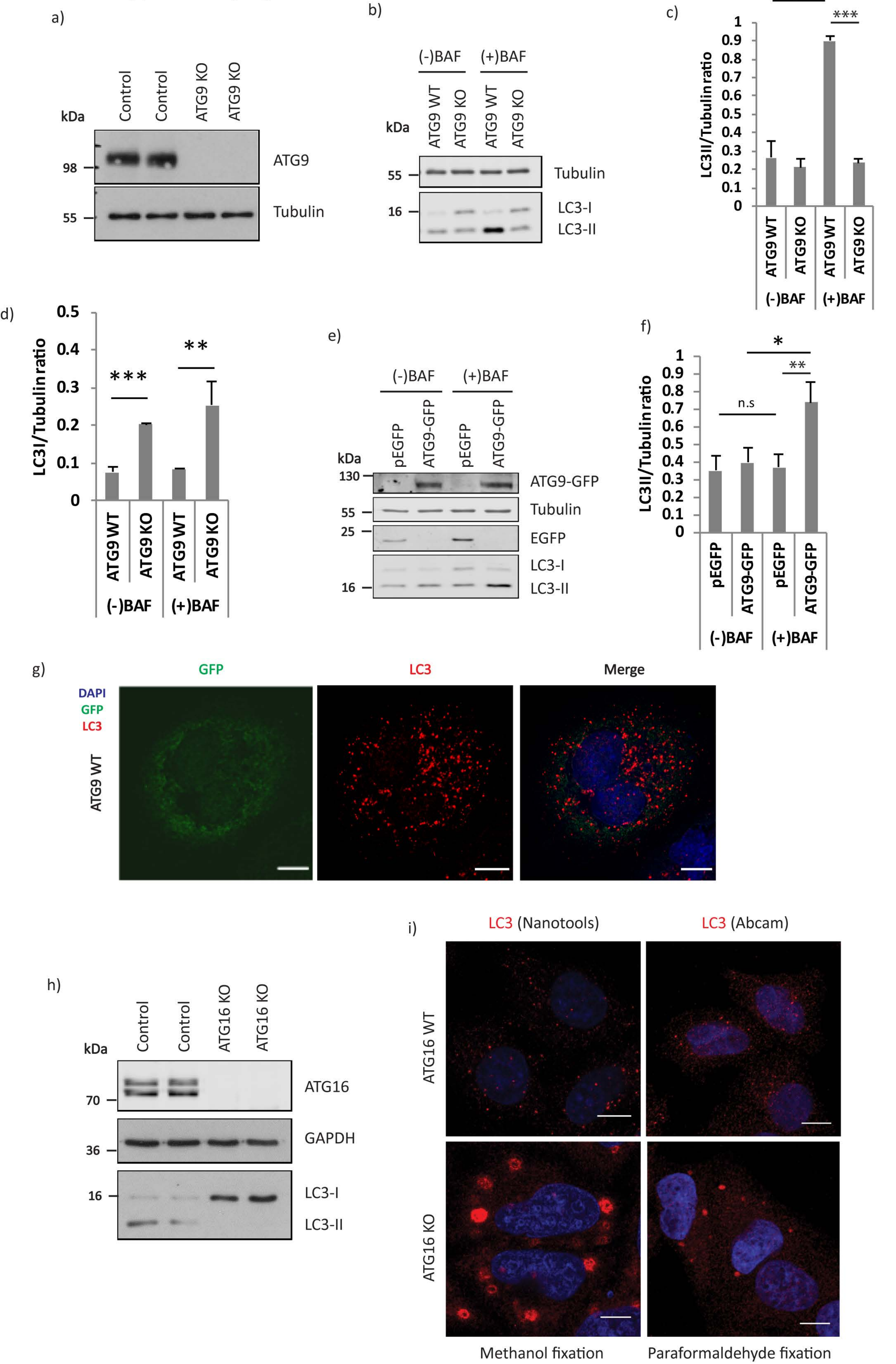

Supplementary figure 2

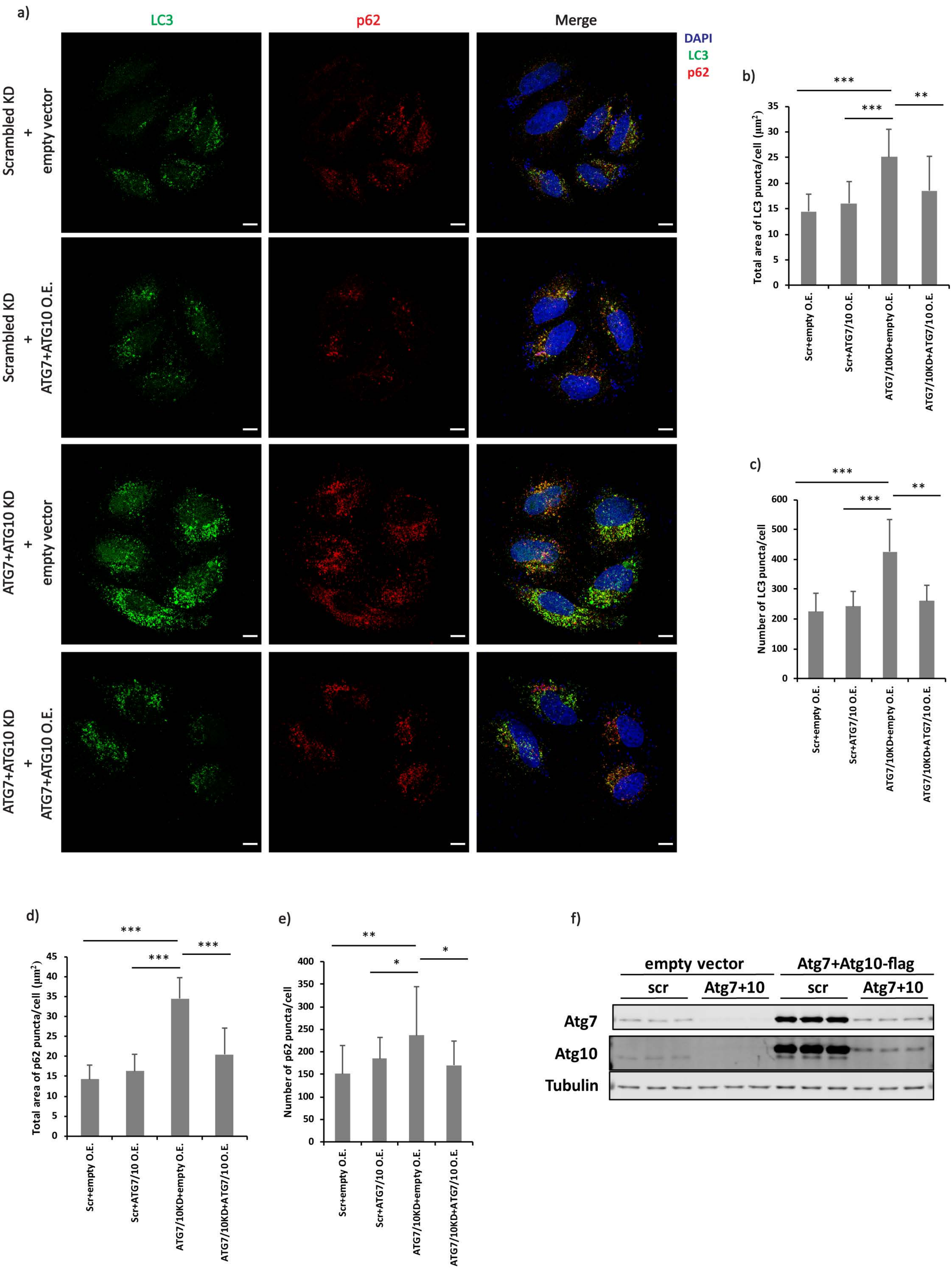

Supplementary Figure 3

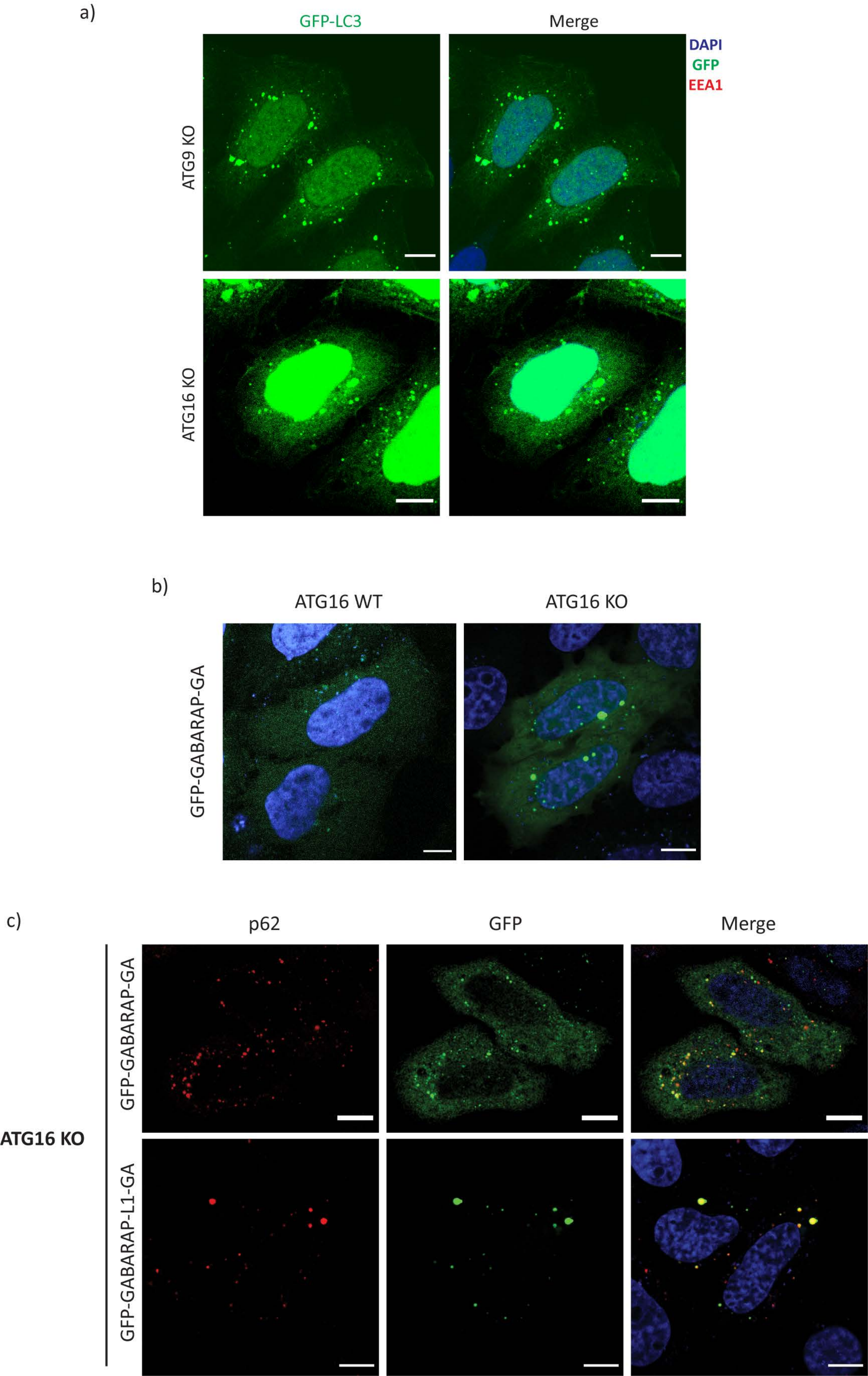

Supplementary Figure 4

ATG9 KO cells

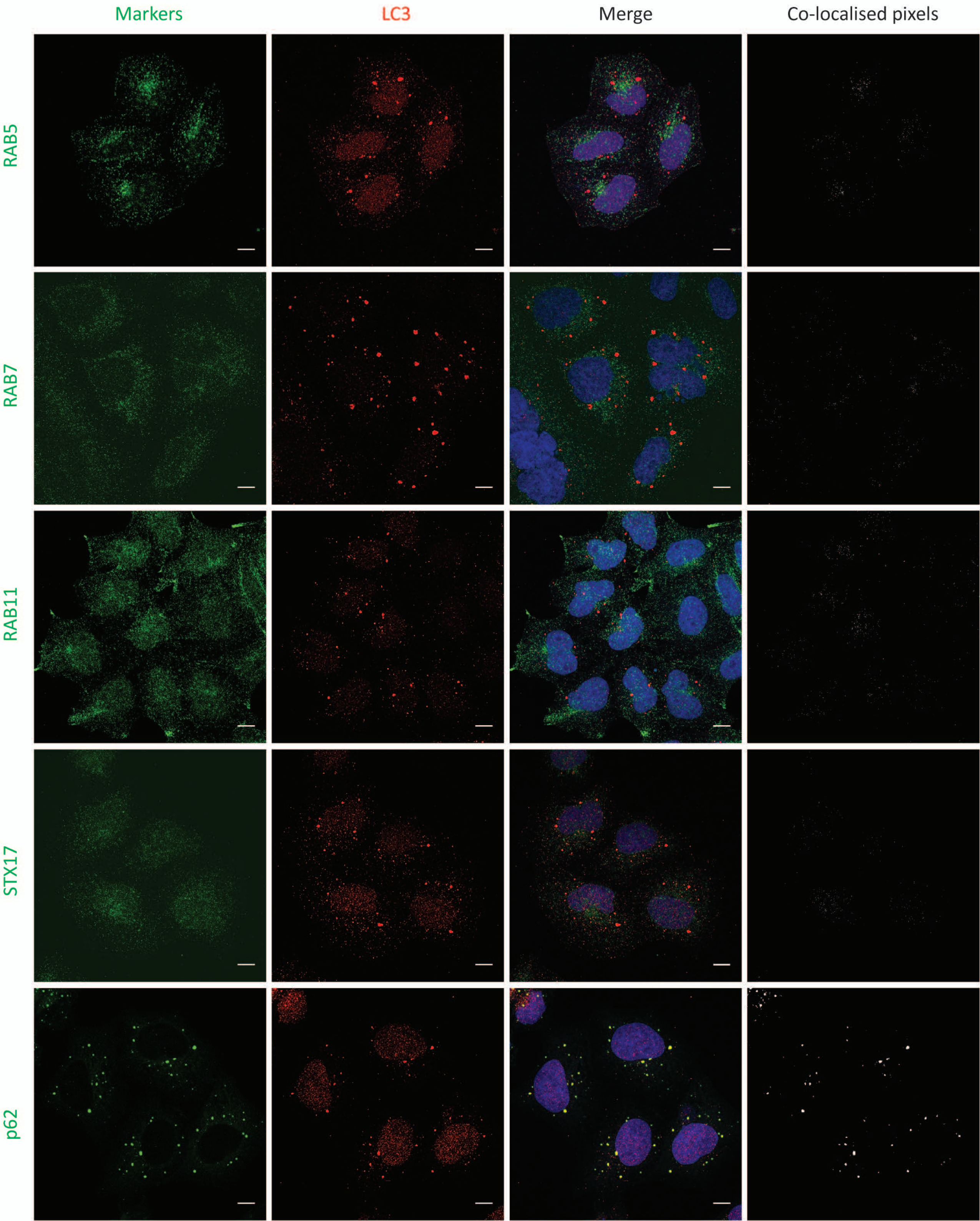

Supplementary Figure 5

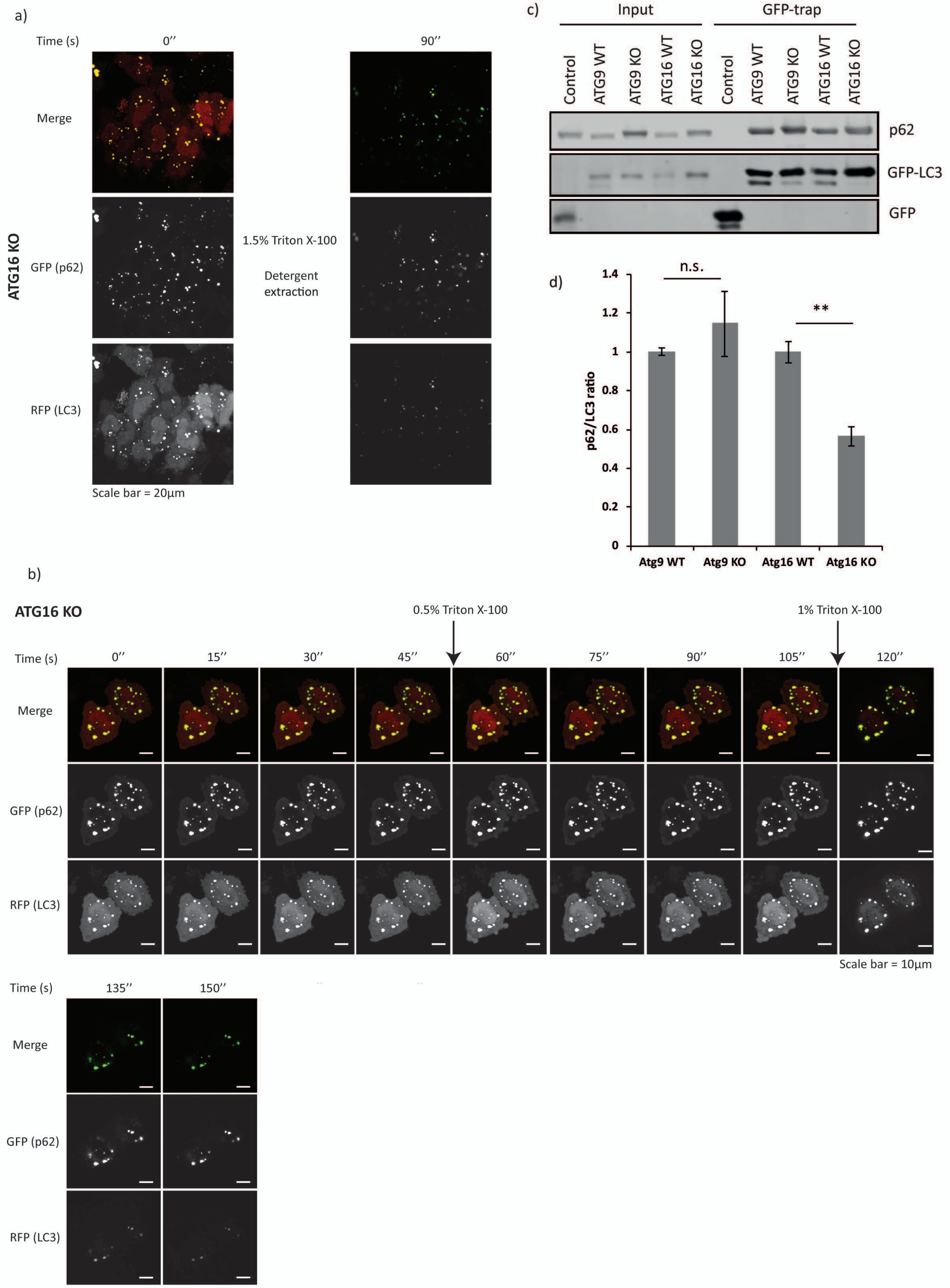

Supplementary Figure 6

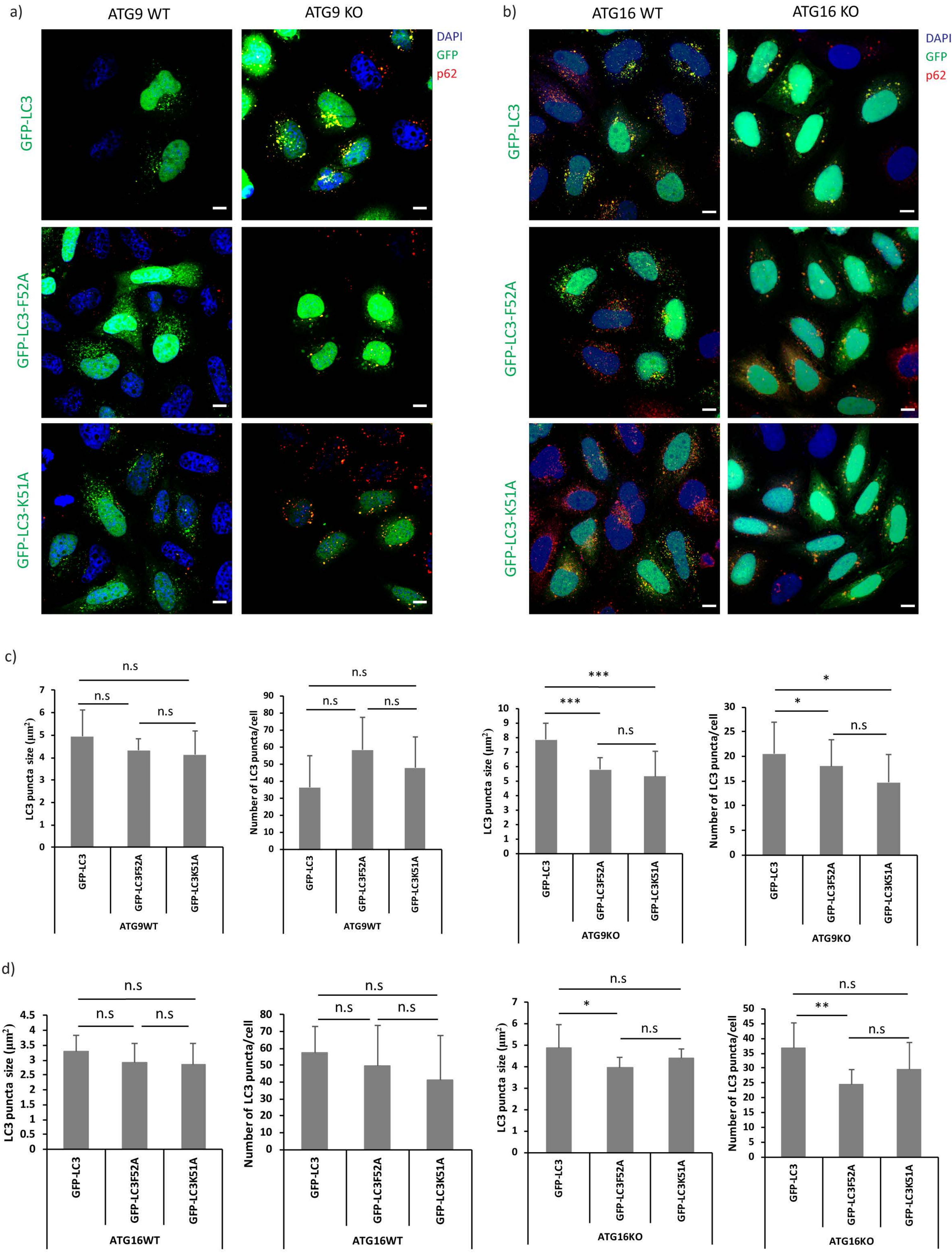

Supplementary Figure 7

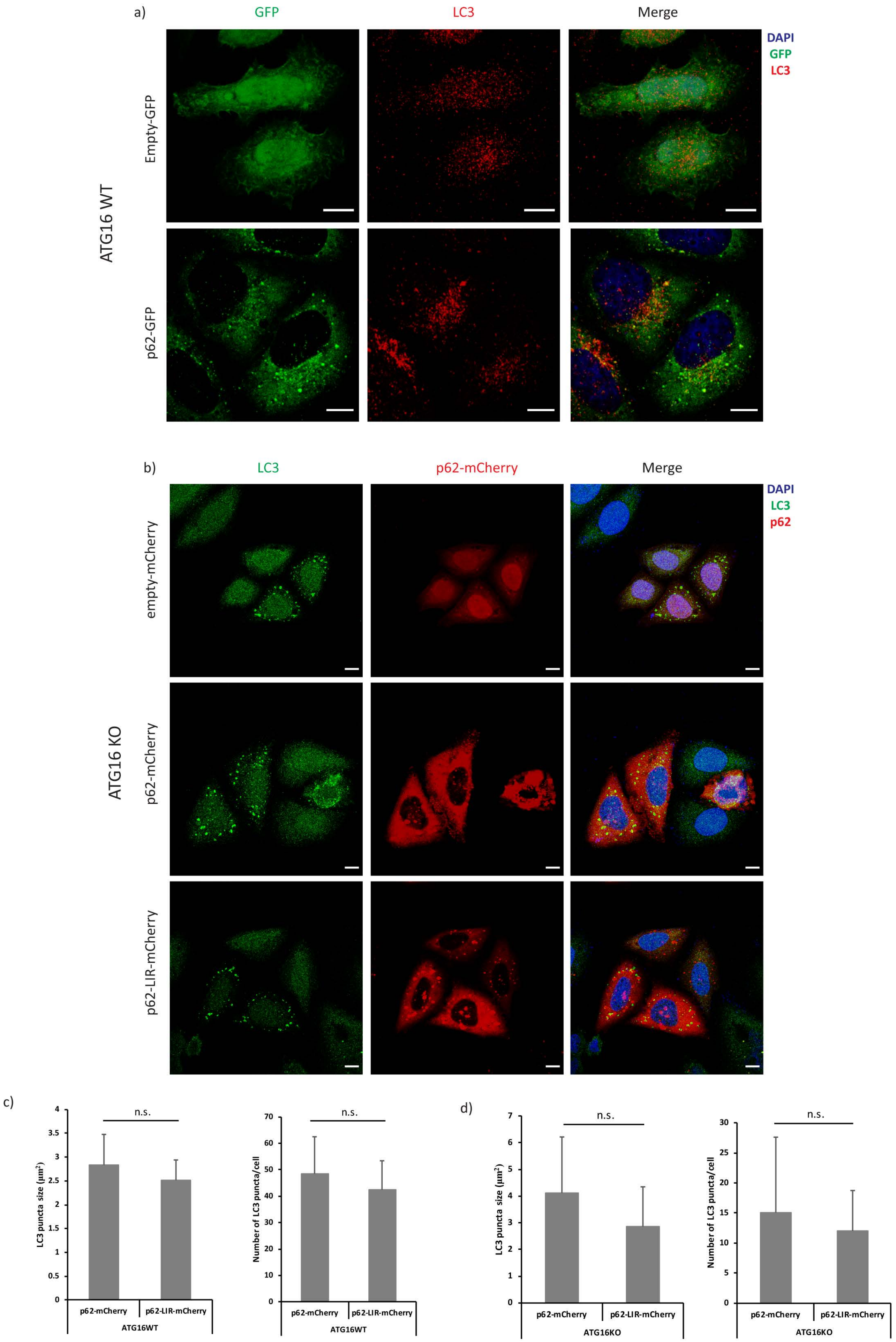

Supplementary Figure 8

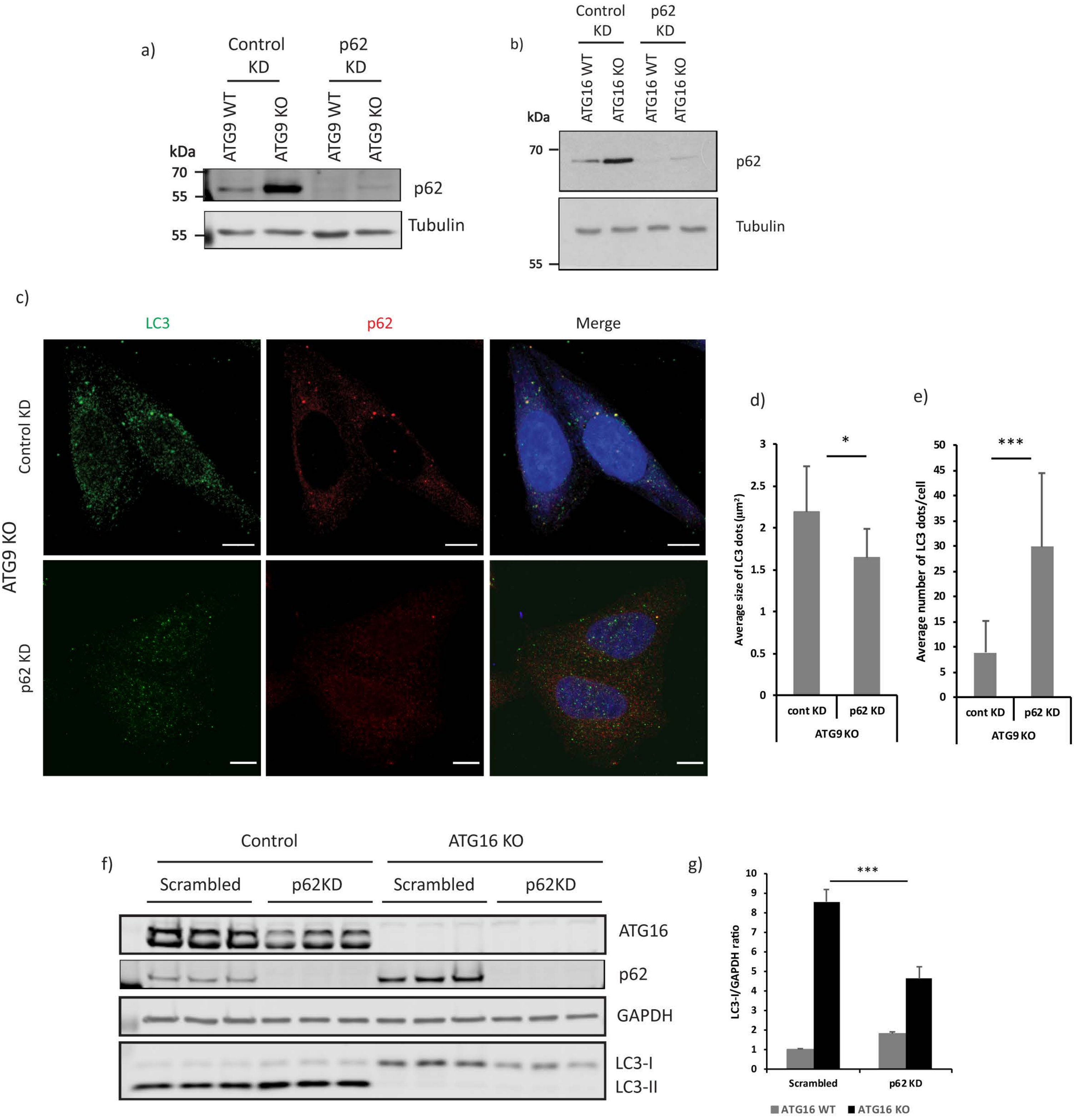

Supplementary Figure 9

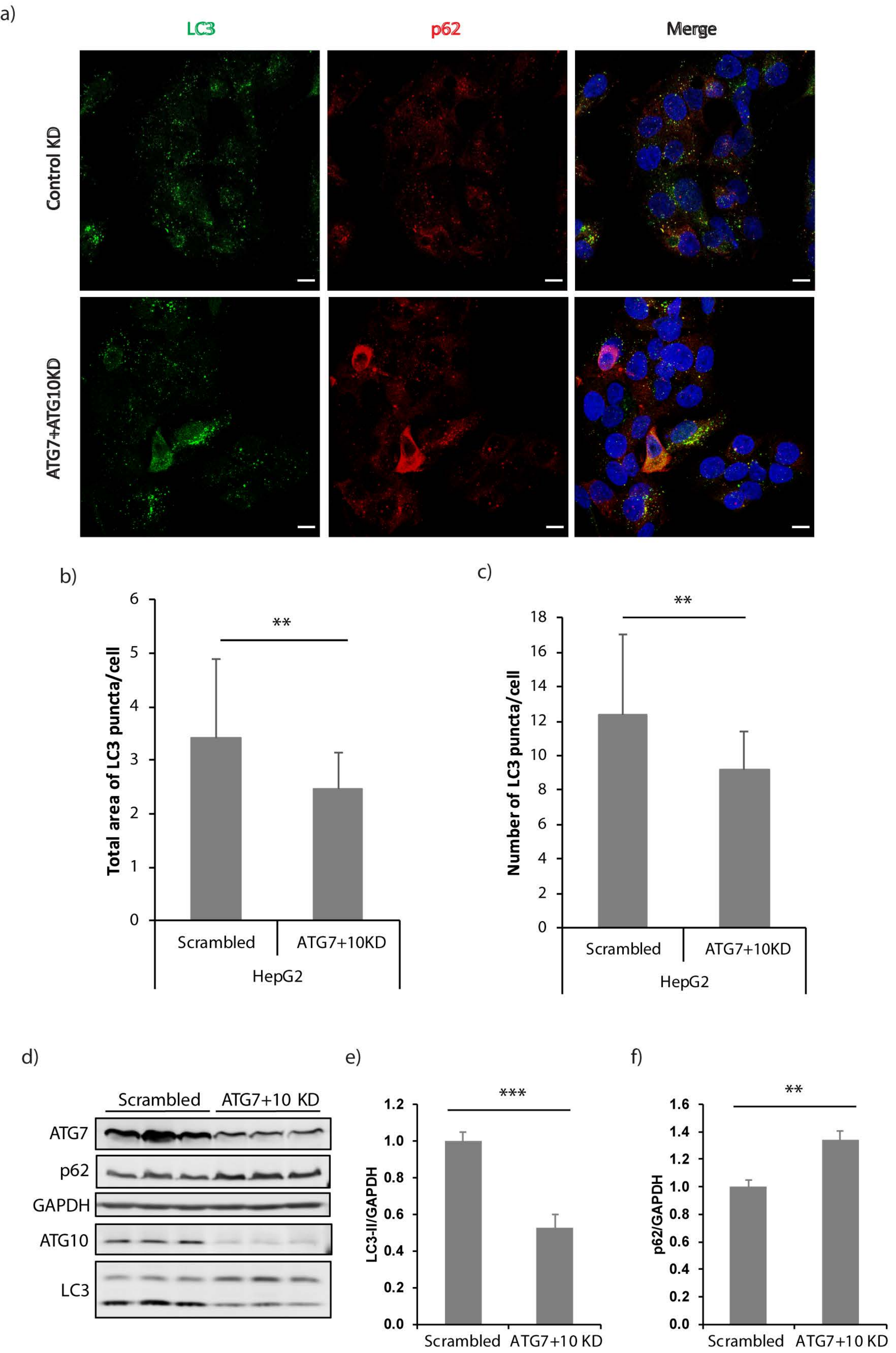

Full-length gels/blots

Blots associated to Figure 2

Fig 2a

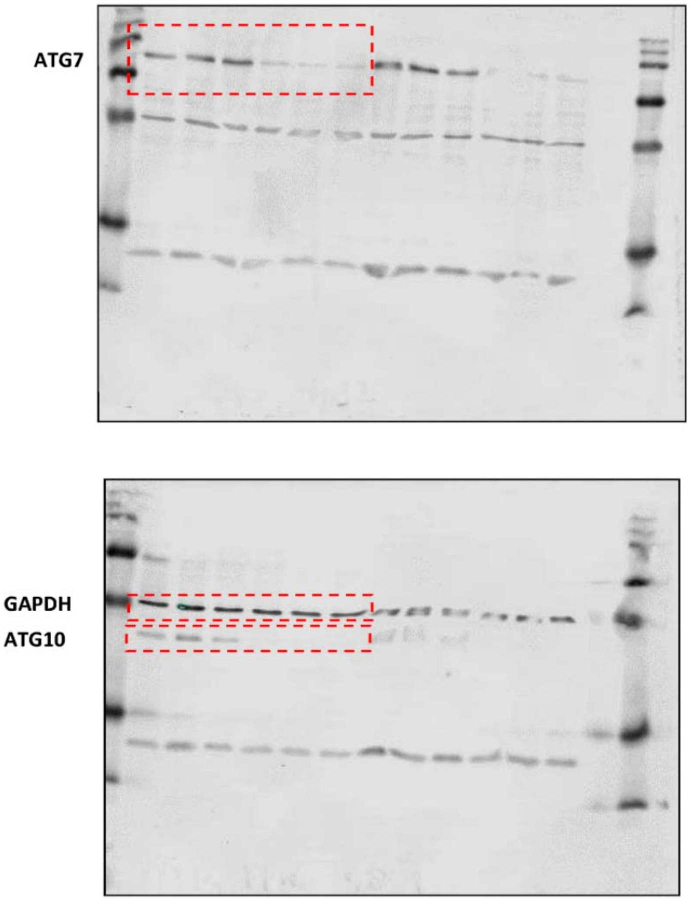

Fig 2b

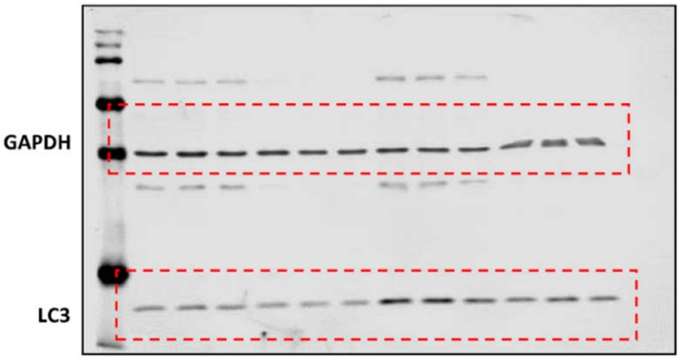

Blots associated to Figure 6

Fig 6b

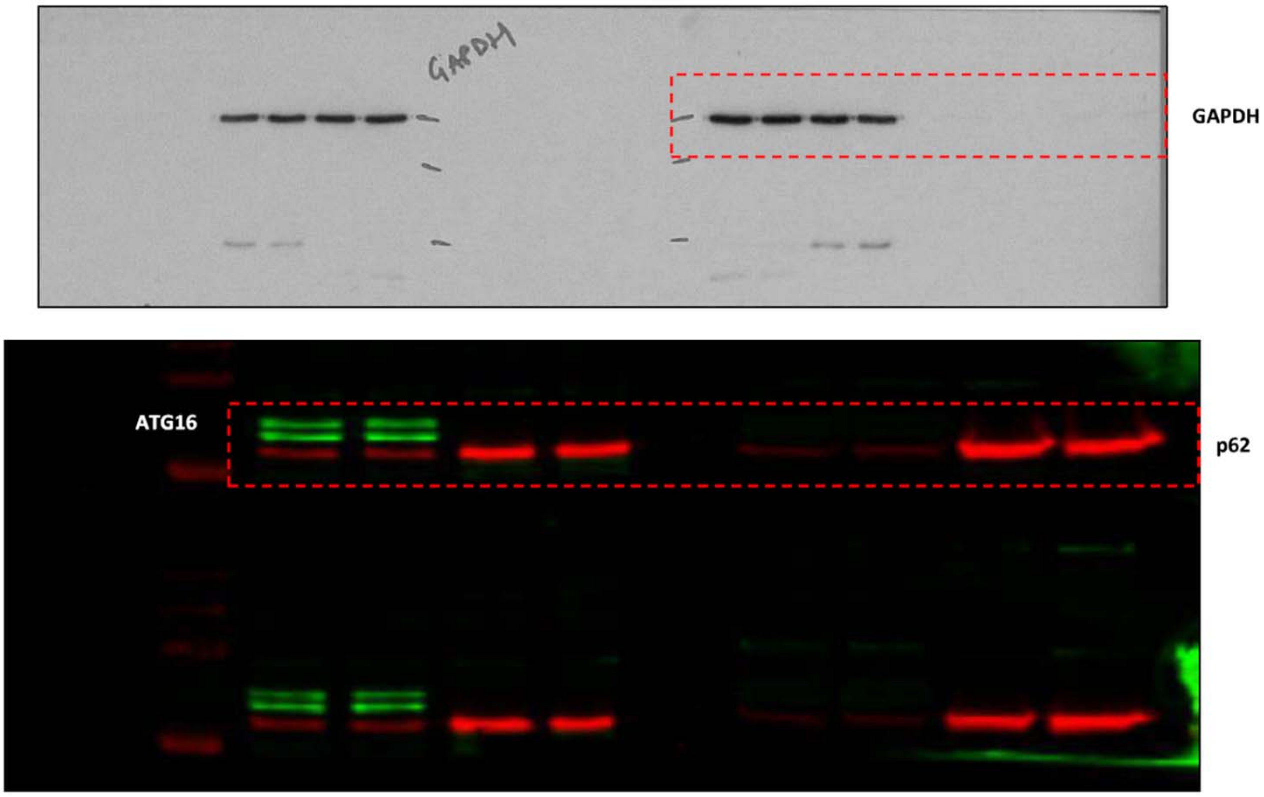

Blots associated to Supplementary Figure 1

Supp Fig 1a

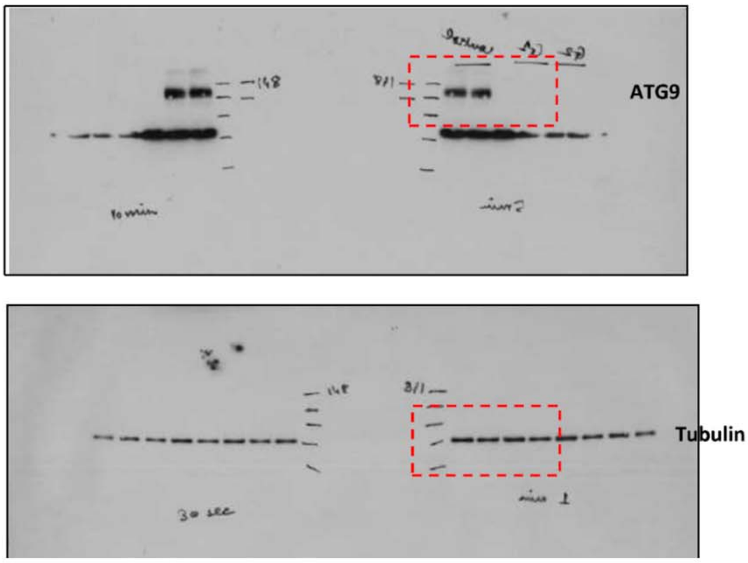

Supp Fig 1b

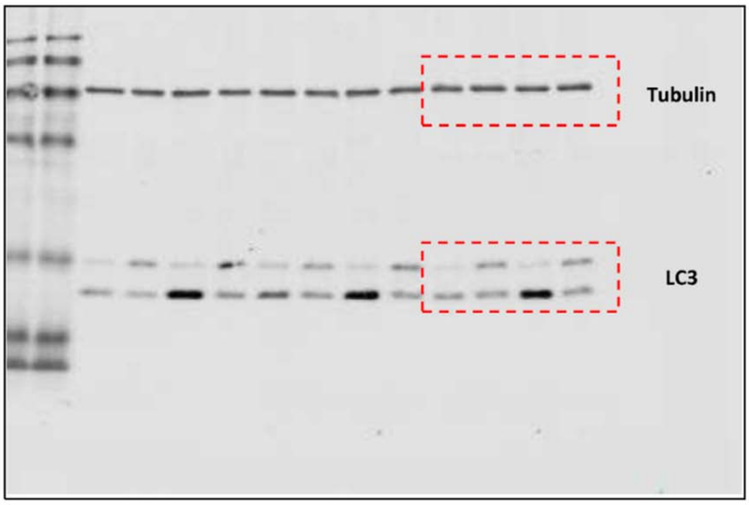

Supp Fig 1e

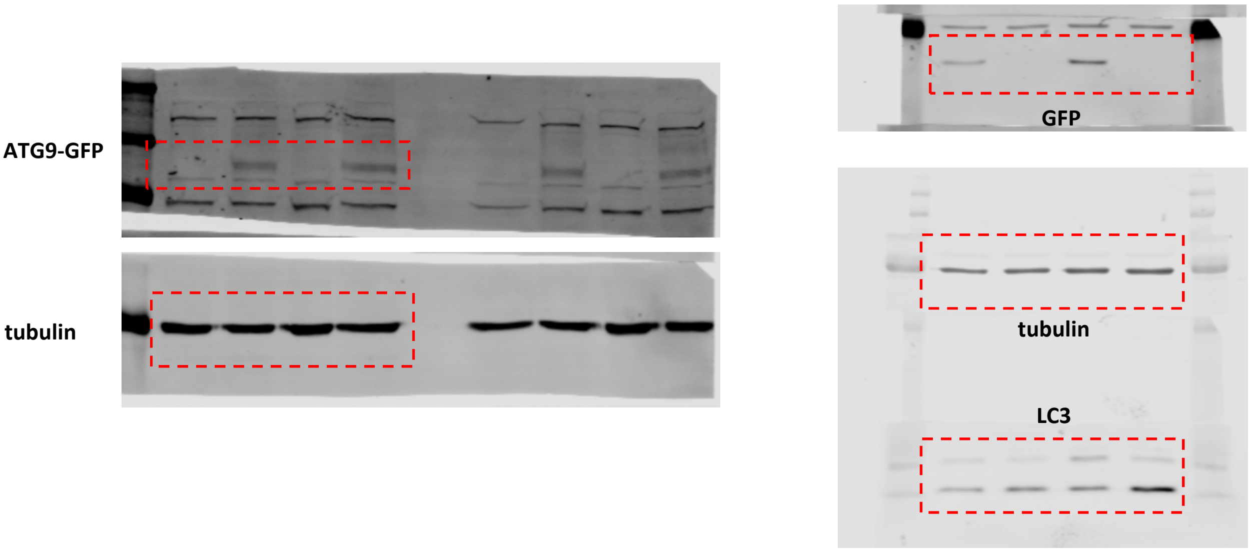

Blots associated to Supplementary Figure 1 (cont.)

Supp Fig 1g

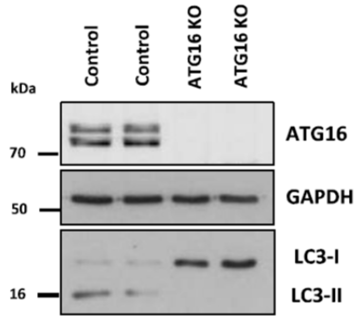

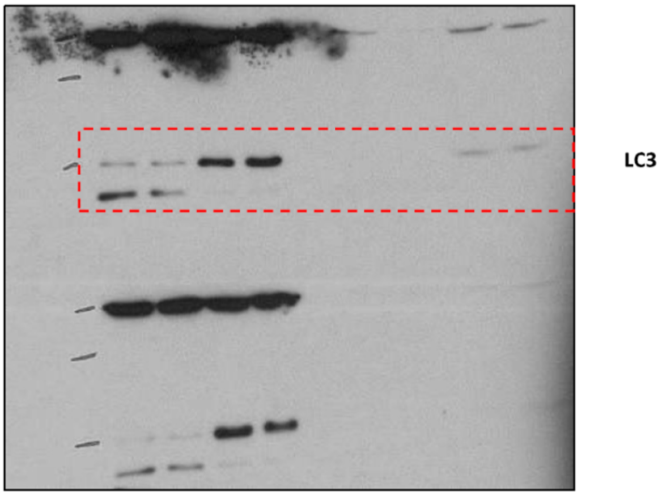

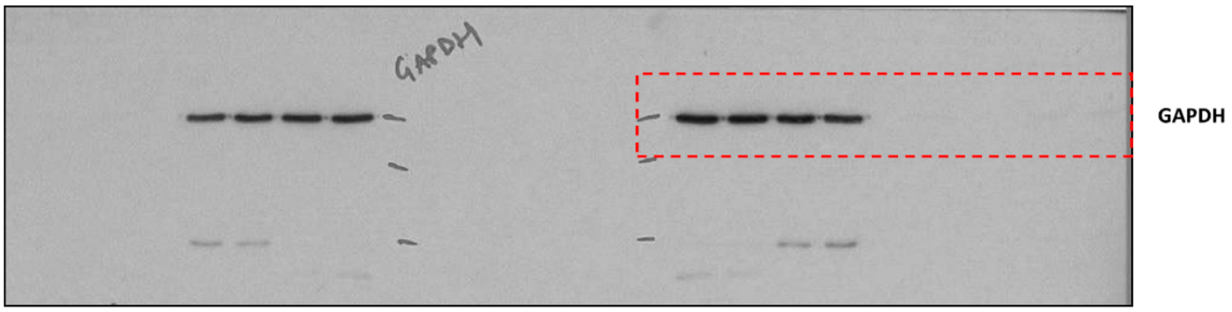

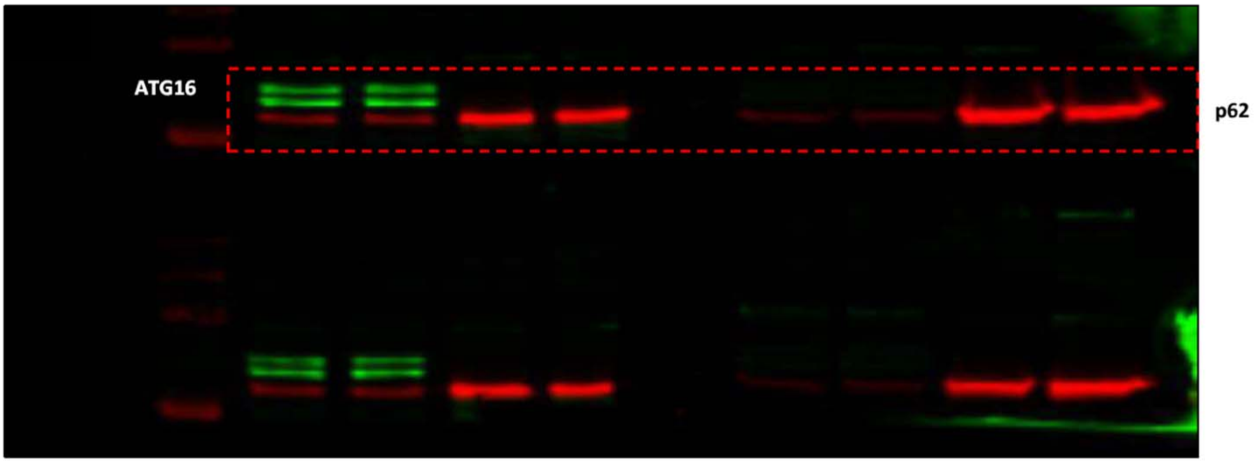

Blots associated to Supplementary Figure 2

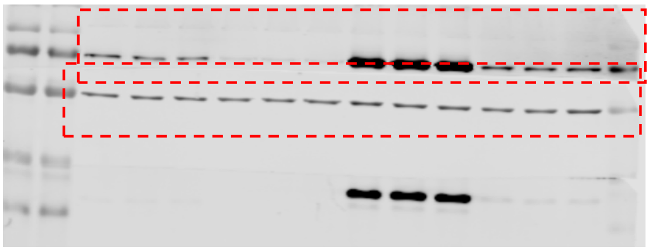

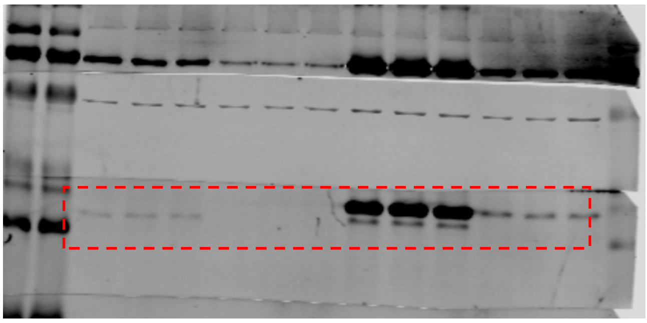

Blots associated to Supplementary Figure 8

Supp Fig 8a

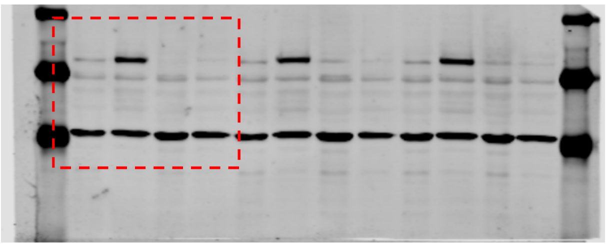

Supp Fig 8b

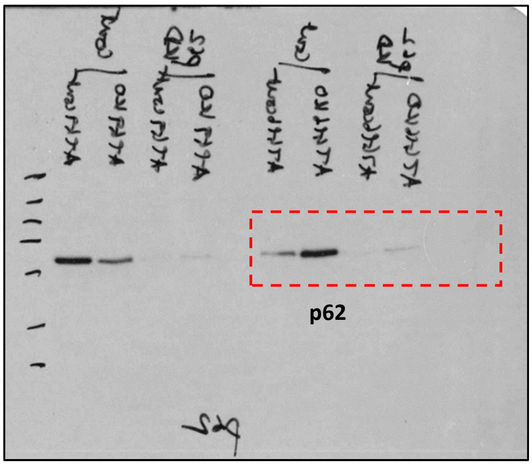

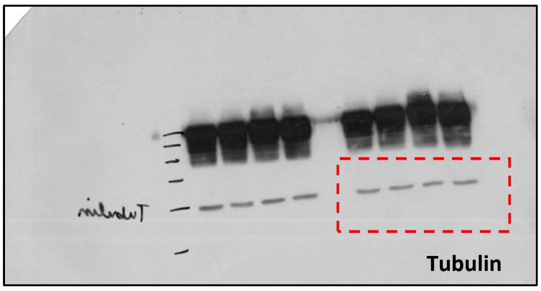

Supp Fig 8f

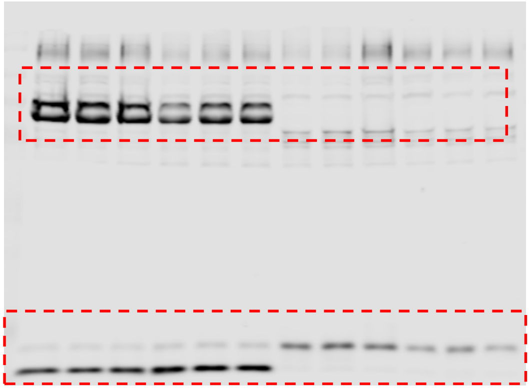

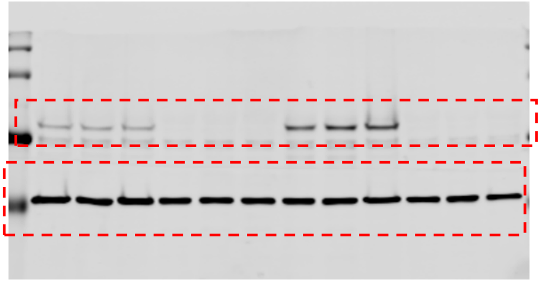

Blots associated to Supplementary Figure 9

Supp Fig 9d

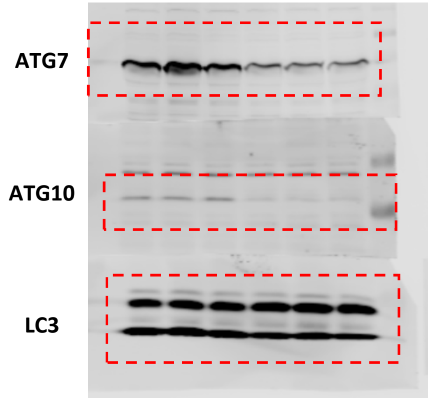

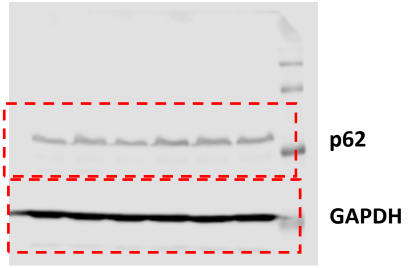

Supplement: Supplementary file 1 — Supplementary Figures and legends and uncropped blots [file 41598_2019_46657_MOESM1_ESM.pdf]
